# A Unified Approach to Synthesizing Four Linezolid Metabolites That May Cause Thrombocytopenia

**DOI:** 10.3390/ph18121821

**Published:** 2025-11-28

**Authors:** Naoki Oikawa, Natsu Inoue, Shogo Ishii, Takumi Goto, Hiroaki Saito, Fumihiro Kurosaki, Takahiro Aoyama, Yasuhiro Tsuji, Taketo Uchiyama

**Affiliations:** 1 Laboratory of Medicinal Chemistry, School of Pharmacy, Nihon University, 7-7-1, Narashinodai, Funabashi 274-8555, Chiba, Japan; phna17028@g.nihon-u.ac.jp (N.I.); shogo.i.1120@gmail.com (S.I.); g.takumi.0315@gmail.com (T.G.); saito.hiroaki@nihon-u.ac.jp (H.S.); 2 Laboratory of Clinical Pharmacometrics, School of Pharmacy, Nihon University, 7-7-1, Narashinodai, Funabashi 274-8555, Chiba, Japan; kurosaki.fumihiro@nihon-u.ac.jp (F.K.); aoyama.takahiko@nihon-u.ac.jp (T.A.); tsuji.yasuhiro@nihon-u.ac.jp (Y.T.)

**Keywords:** linezolid, metabolites, synthesis, PNU-142618, PNU-142300, PNU-142586, PNU-173558, Ns strategy

## Abstract

**Background/Objectives**: Linezolid is a first-in-class oxazolidinone antibiotic that exhibits activity against Gram-positive pathogens, including methicillin-resistant *Staphylococcus aureus* and vancomycin-resistant enterococci. However, its clinical use is often restricted because of hematological toxicities, particularly thrombocytopenia, in patients with renal impairment. That side effect is thought to result from the systemic accumulation of pharmacologically inactive metabolites generated by oxidative degradation and ring-opening of the morpholine, but the details remain unclear. In this study, we established a novel synthetic route for four linezolid metabolites (PNU-142618, 142300, 142586 and 173558). **Methods**: The four major metabolites, which are secondary or tertiary amines, were synthesized using the aniline derivatives protected with a 2-nitrobenzensulfonyl (Ns) group. **Results**: Application of this Ns strategy enabled selective *N*-alkylation, enabling efficient synthesis of the target metabolites. The desired metabolites containing a carboxylic acid group were obtained as their sodium salts. This is the first report on the synthesis of PNU-142618 and 173558. **Conclusions**: The established synthetic pathway provides access to four linezolid metabolites. The results facilitated the provision of compounds necessary for comprehensive pharmacokinetic and toxicological studies.

## 1. Introduction

Linezolid is an oxazolidinone antibiotic that exhibits broad-spectrum activity against Gram-positive bacteria, including methicillin-resistant *Staphylococcus aureus* and vancomycin-resistant enterococci [1]. It has been approved to treat various infections caused by multidrug-resistant organisms and has demonstrated superior efficacy to other antibiotics, such as quinupristin–dalfopristin. The antibacterial mechanism of action involves selective binding to the 50S ribosomal subunit, which inhibits the formation of the 70S initiation complex and inhibits bacterial protein synthesis.

**Figure 1:**
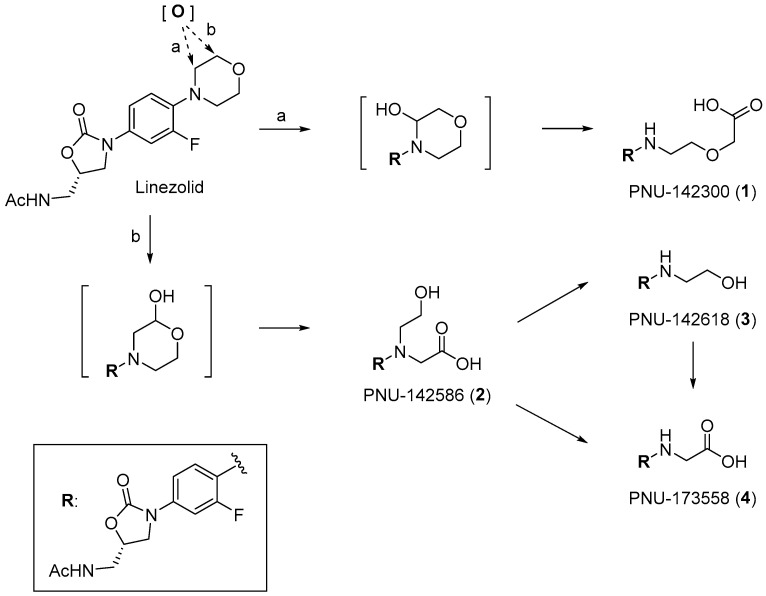
Proposed metabolic pathway of linezolid.

Despite its clinical effectiveness, linezolid treatment is associated with the incidence of hematological adverse events, particularly thrombocytopenia, which prevents its long-term use. These toxicities are more pronounced in patients with renal impairment, which has prompted the re-evaluation of dosing strategies based on renal function [2]. Linezolid-induced thrombocytopenia may be caused by the accumulation of its major inactive metabolites, PNU-142300 (**1**) and PNU-142586 (**2**) [3,4,5,6]. These are generated by oxidative cleavage of the morpholine ring on linezolid through routes a and b, respectively, and are primarily eliminated through the kidneys. And these metabolites are known to be P450-independent [7,8]. Furthermore, metabolites such as PNU-142618 (**3**) and PNU-173558 (**4**) may also form from PNU-142300 (**1**) and/or PNU-142586 (**2**), although the mechanism of their metabolic pathways remains unclear, and toxicological evaluations have not yet been conducted (Figure 1, see Supplementary Materials) [9].

It was also suggested that PNU-142586 (**2**) may cause myelosuppression by exerting a concentration-dependent cytotoxic effect on megakaryocytic cells. In addition, the transport of this metabolite via human organic anion transporter 3 (hOAT3) and its inhibition by other drugs, such as proton pump inhibitors, may further exacerbate its accumulation [10]. The complexity of linezolid metabolism and interindividual variability in renal clearance highlight the need for methods to precisely measure and evaluate parent drug and metabolite concentrations in the plasma and urine [11,12]. So, advanced analytical techniques such as UPLC–MS/MS have been established to facilitate these pharmacokinetic analyses in healthy individuals and individuals with impairment in hepatic/renal function [6,13].

Although the primary metabolic pathways of linezolid have been partially elucidated, the importance of pharmacokinetics for linezolid metabolites and their toxicology remains unclear [14,15]. One of the reasons for this limitation is the difficulty in acquiring the metabolites as standard materials for the studies. The synthesis of linezolid metabolites is necessary for elucidating the mechanisms underlying linezolid-associated adverse effects. Therefore, synthetic strategies to prepare these metabolites are important, but to date, only one paper has been published on the chemical synthesis of linezolid metabolites PNU-142300 (overall yield 23%) and PNU-142586 (overall yield 7%) by Hanaya and Sugai et al. [16]. In this study, we adopted the 2-nitrobenzenesulfonyl (Ns)-strategy developed by Kan and Fukuyama [17,18], which enables efficient protection, selective *N*-alkylation, and mild deprotection of amines, thereby enabling the introduction of selective alkyl chains onto aniline derivatives.

## 2. Results and Discussion

### 2.1. Retrosynthetic Analysis

**Figure 2:**
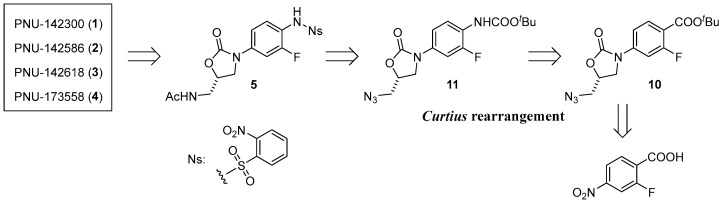
Retrosynthetic analysis for the synthesis of linezolid metabolites **1**–**4**.

Since the desired linezolid metabolites are aromatic secondary or tertiary amine derivatives, we incorporated the Ns group into the designed synthetic route, which is useful as a protective group for amines, and adopted compound **5** as a common intermediate [19]. Compound **5** can be prepared from carbamate obtained by *Curtius* rearrangement reaction. And the *^t^*Bu esters used in *Curtius* rearrangement reaction were also obtained from 2-fluoro-4-nitrobenzoic acid as starting material (Figure 2).

### 2.2. Preparation of the Common Intermediate ***5***

**Scheme 1:**
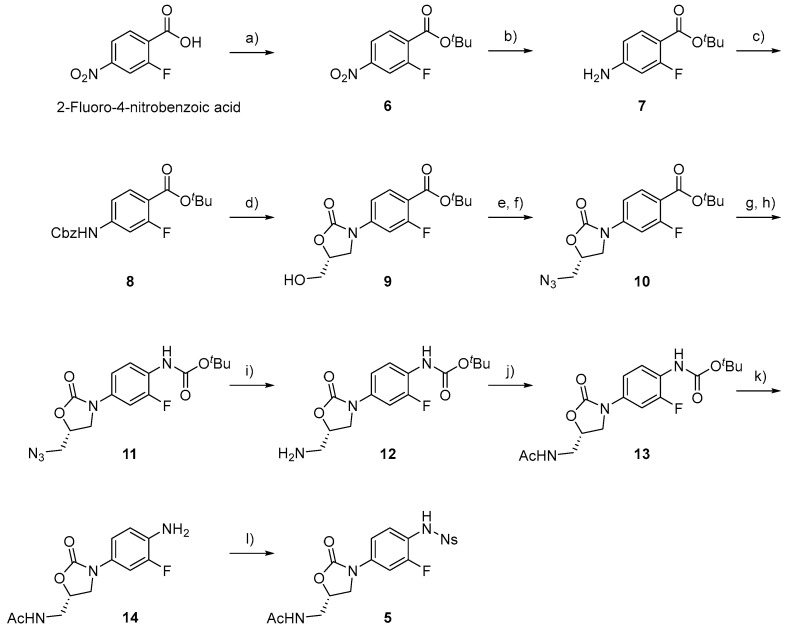
Synthesis of the common intermediate **5**. (a) TEA, DMAP, Boc_2_O, DCM, rt, 2 h (83%). (b) H_2_, Pd/C, MeOH, rt, 2 h (quant). (c) Cbz-Cl, Py, rt, 18 h (99%). (d) *R*-(-)-Glycidyl butyrate, *n*-BuLi, THF, -78 °C, 18 h (71%). (e) TEA, Ms-Cl, DCM, rt, 1 h. (f) NaN_3_, DMF, 60 °C, 4 h (82%, 2 steps). (g) TFA, DCM, rt, 1 h. (h) *t*-BuOH, TEA, DPPA, THF, 70 °C, 4 h (56%, 2 steps). (i) H_2_, Pd/C, MeOH, rt, 1 h (quant). (j) TEA, Ac_2_O, DCM, rt, 0.5 h (quant). (k) TFA, DCM, rt, 1 h. (l) NsCl, Py, rt,1.5 h (2 steps, 92%).

2-Fluoro-4-nitrobenzoic acid was treated with *tert*-butoxycarbonyl (Boc) anhydride to obtain the corresponding *tert*-butyl ester **6**. Next, catalytic hydrogenation of **6** afforded the aniline derivative **7**, whose primary amine group was then treated with benzyl chloroformate (Cbz-Cl) to give the carbamate **8**. The oxazolidinone derivative **9** was yielded with **8** and a chiral glycidyl ester under *n*-butyllithium conditions based on Manninen and Brickner’s procedure [1,19]. Resulting primary alcohol on **9** was substituted with azide group via mesylate to give **10**. *tert*-Butyl ester derivative **10** was treated with trifluoroacetic acid (TFA) to afford carboxylic acid without further purification, followed by *Curtius* rearrangement reaction to obtain aniline derivative **11**. Using catalytic hydrogenation for azide group on **11** afforded the corresponding primary amine **12**, followed by acetylation with acetic anhydride (Ac_2_O) to yield **13**. Then, **13** was treated with TFA to eliminate the Boc group, yielding the aniline derivative **14**. Finally, treatment of **14** with 4-nitrobenzenesulfonyl chloride in pyridine gave the desired common intermediate **5** (overall yield: 25%, 12 steps) (Scheme 1).

### 2.3. Synthesis of PNU-142300 (***1***)

**Scheme 2:**
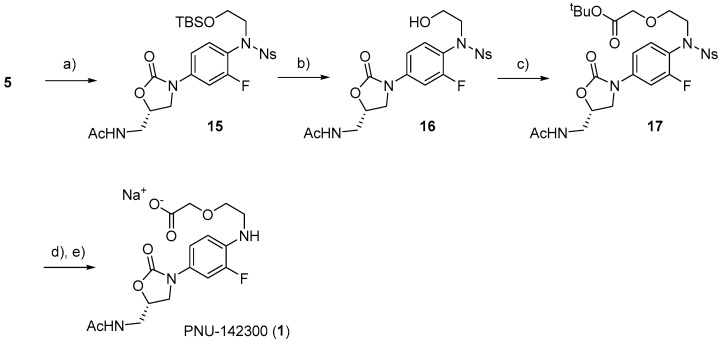
Synthesis of PNU-142300 (**1**). (a) (2-Bromoethoxy)-*tert*-butyldimethylsilane, K_2_CO_3_, DMF, 50 °C, 48 h (86%). (b) TBAF, THF, rt, 2 h (85%). (c) *tert*-Butyl bromoacetate, 1 M NaOH, *^n^*Bu_4_NHSO_4_, DCM, rt, 2 h (77% based on conversion yield, 43% recovered **16**). (d) Thiophenol, K_2_CO_3_, DMF, rt, 6 h (86%). (e) TFA, DCM, rt, 1 h, 1 M NaOH (94%).

The Ns-protected intermediate **5** was treated with (2-bromoethoxy)-*tert*-butyldimethylsilane in *N*,*N*-dimethylformamide (DMF) in the presence of potassium carbonate, which yielded compound **15**. Deprotection of the *tert*-butyldimethylsilyl group with tetrabutylammonium fluoride (TBAF) yielded the primary alcohol derivative **16**. *O*-Alkylation of **16** was carried out under biphasic conditions using tetrabutylammonium hydrogen sulfate as a phase-transfer catalyst according to the procedure of Sugai et al. [16] to obtain **17.** In this *O*-alkylation reaction, the reaction proceeded as intended and the desired compound **17** was obtained with 77% (conversion yield), but the reaction was incomplete (recovered **16** with 43%). Finally, Ns group of **17** was removed with thiophenol, followed by deprotection of the *tert*-butyl group in **17** using TFA, and neutralization with 1 M sodium hydroxide solution to yield a sodium salt of PNU-142300 (**1**) (overall yield: 11%, 17 steps) (Scheme 2).

### 2.4. Synthesis of PNU-142586 (***2***)

**Scheme 3:**
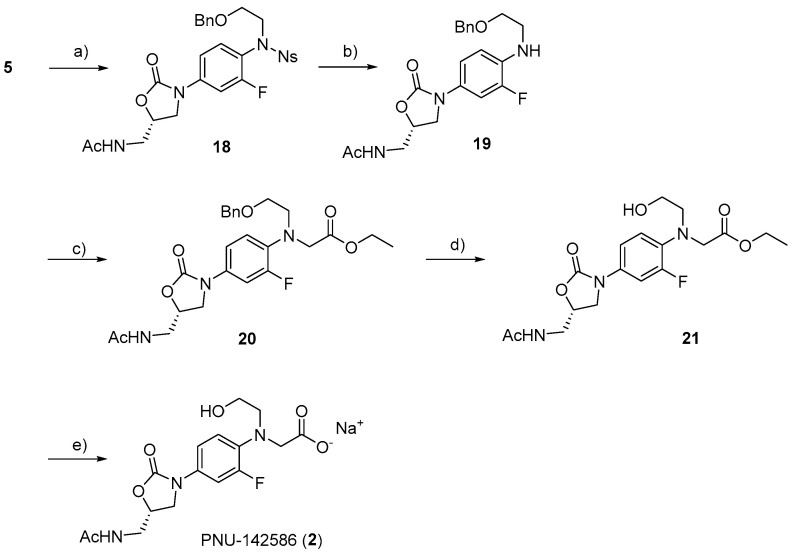
Synthesis of PNU-142586 (**2**). (a) Benzyl 2-bromoethyl ether, K_2_CO_3_, DMF, 50 °C, overnight (90%). (b) Thiophenol, K_2_CO_3_, DMF, rt, 6 h (quant). (c) Ethyl bromoacetate, K_2_CO_3_, MeCN 90 °C, 36 h (68%). (d) Pd/C, H_2_, THF rt, 24 h (quant). (e) 1 M NaOH, THF, rt, 2 h (80%).

A common intermediate **5** was alkylated with a benzyl 2-bromoethyl ether to give **18**, followed by the removal of the Ns group using thiophenol to afford secondary amine **19**. The *N*-alkylation reaction of **19** treated with ethyl bromoacetate in the presence of potassium carbonate to obtain **20** required high temperatures and extended reaction time to improve yield. Then, the benzyl group on **20** was removed by hydrogenation over Pd/C to afford primary alcohol **21**. Then, **21** in THF was treated with aqueous sodium hydroxide to hydrolyze the ester, which afforded **2** with precipitation as sodium salt (overall yield: 12%, 17 steps) (Scheme 3).

### 2.5. Synthesis of PNU-142618 (***3***)

**Scheme 4:**
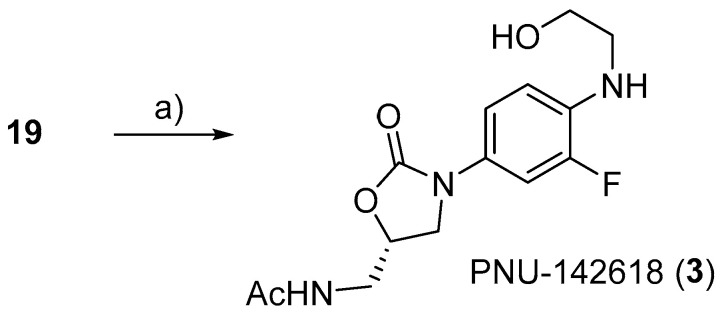
Synthesis of PNU-142618 (**3**). (a) Pd/C, H_2_, THF rt, 24 h (86%).

PNU-142618 (**3**) was obtained by debenzylation with Pd/C and H_2_ of **19** (overall yield: 22%, 15 steps) (Scheme 4).

### 2.6. Synthesis of PNU-173558 (***4***)

**Scheme 5:**
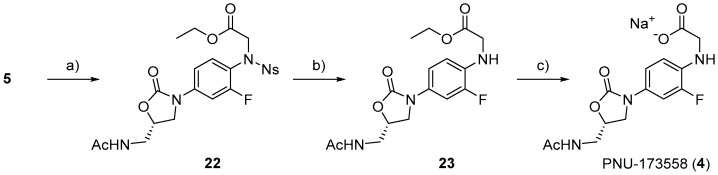
Synthesis of PNU-173558 (**4**). (a) Ethyl bromoacetate, K_2_CO_3_, DMF 50 °C, overnight (95%). (b) Thiophenol, K_2_CO_3_, DMF, rt, 6 h. (c) 1 M NaOH, THF, rt, 2 h (95%, 2 steps).

Analogous to compound **2**, PNU-173558 (**4**) was also designed for preparation as the sodium salt. Specifically, compound **4** was synthesized by *N*-alkylation of the common intermediate **5** with ethyl bromoacetate and potassium carbonate to afford **22**, followed by deprotection of the Ns group using thiophenol, and ester hydrolysis with 1 M NaOH (overall yield: 23%, 15 steps) (Scheme 5).

## 3. Materials and Methods

### 3.1. General Remarks

All reagents and solvents were of the highest commercial grade and used without further purification. ^1^H-NMR spectra were measured at 600 MHz on a JEOL JNM-ECX600 spectrometer (JOEL, Tokyo, Japan). Chemical shifts are reported relative to internal standards (tetramethylsilane, δH 0.00, CDCl_3_, δH 7.26, CD_3_OD, δH 3.31). Data are presented as follows: chemical shift (δ, ppm), multiplicity (s = singlet, d = doublet, t = triplet, q = quartet, and m = multiplet), coupling constant, and integration. ^13^C-NMR spectra were measured at 151 MHz using a JEOL JNM-ECX600 spectrometer. The following internal references were used: tetramethylsilane: δ 0.00, CDCl_3_ δ 77.0, CD_3_OD, δ 49.0). High-resolution mass spectra were recorded using a Waters Xevo G2-S QTOF instrument (Waters, Milford, MA, USA). Silica gel column chromatography was done using Silica Gel 60N (spherical and neutral, particle size 40 to 100 µm, Kanto Chemical Co., Tokyo, Japan). Analytical thin-layer chromatography was conducted using Merck silica gel 60 F_254_ plates with visualization by ultraviolet light or spraying with Ninhydrin–ethanol test solution spray on a hot plate. Preparative thin-layer chromatography was performed using Merck silica gel 60 F_254_ plates with visualization by ultraviolet light. The high-performance liquid chromatography system (HPLC) consisted of a PU-1580 (JASCO, Tokyo, Japan), SSC-5410 (Senshu Scientific Co., Ltd., Tokyo, Japan), and column (SG80, 5 μm, 20 mm I.D. × 250 mm). Optical rotations were measured on a JASCO P-1030 digital polarimeter at the sodium D line (589 nm). Melting points were measured on an ATM-01 (AS ONE Co., Osaka, Japan).

### 3.2. Synthesis

#### 3.2.1. *tert*-Butyl 2-fluoro-4-nitrobenzoate (**6**) [20]

To a solution of 2-fluoro-4-nitrobenzoic acid (6.0 g, 32 mmol) in dichloromethane (DCM) (60 mL), triethylamine (TEA) (13 mL, 93 mmol), dimethylaminopyridine (DMAP) (1.2 g, 9.8 mmol), and di-*tert*-butyl dicarbonate (Boc_2_O) (11 mL, 49 mmol) were added. After stirring for 2 h at room temperature, the residue was treated with ethyl acetate (EtOAc) and 5% citric acid solution. The organic layer was washed with brine and dried over anhydrous Na_2_SO_4_. The filtrate was concentrated under reduced pressure to obtain a colorless solid **6** (6.6 g, 83%). M.P. 81–82 °C. ^1^H-NMR (600 MHz, CDCl_3_) δ 8.07–8.03 (m, 2H), 7.99–7.97 (m, 1H), 1.62 (s, 9H).

#### 3.2.2. *tert*-Butyl 4-amino-2-fluorobenzoate (**7**) [20]

To a solution of **6** (1.1 g, 5.0 mmol) in methanol (MeOH) (30 mL), Pd/C (200 mg) was added. After stirring for 2 h under a H_2_ atmosphere at room temperature, the mixture was filtered through a Celite pad. The filtrate was concentrated under reduced pressure, and a colorless solid **7** was obtained (1.0 g, quant.). M.P. 94–96 °C. ^1^H-NMR (600 MHz, CDCl_3_) δ 7.68 (t, *J* = 8.6 Hz), 6.39 (dd, *J* = 2.4, 8.6 Hz), 6.31 (dd, *J* = 2.0, 12.7 Hz), 4.13 (bs, 2H), and 1.57 (s, 9H). HRMS (ESI): *m*/*z* calculated for C_11_H_14_FNNaO_2_: 234.0906, found [M + Na]^+^ 234.0914.

#### 3.2.3. *tert*-Butyl 4-(((benzyloxy)carbonyl)amino)-2-fluorobenzoate (**8**) [21]

To a solution of **7** (3.8 g, 18 mmol) in pyridine (Py) (35 mL), benzyl chloroformate (13 g, 73 mmol) was added dropwise at 0 °C. The mixture was allowed to warm to room temperature. After stirring overnight at room temperature, the mixture was diluted with EtOAc. The residue was treated with EtOAc and 5% hydrochloric acid. The organic layer was washed with brine and dried over anhydrous Na_2_SO_4_. The filtrate was concentrated under reduced pressure. The resulting residue was separated by column chromatography (C.C.) (hexane/EtOAc = 6:1) to yield **8** as a white solid (6.2 g, 99%). M.P. 115–120 °C. ^1^H-NMR (600 MHz, CDCl_3_) δ 7.80 (t, *J* = 8.1 Hz, 1H), 7.41–7.35 (m, 6H), 7.04–6.97 (m, 2H), 5.21 (s, 2H), 1.58 (s, 9H), ^13^C-NMR (151 MHz, CDCl_3_) δ 163.2 (d, *J* = 4.3 Hz), 162.8 (d, *J* = 258.7 Hz), 152.7, 143.0 (d, *J* = 11.6 Hz), 135.5, 132.8, 128.7, 128.6, 128.4, 114.9 (d, *J* = 10.1 Hz), 113.0, 106.3 (d, *J* = 28.9 Hz), and 81.6, 67.5, 28.2. HRMS (ESI): *m*/*z* calculated for C_19_H_20_FNNaO_4_: 368.1274, found [M + Na]^+^ 368.1264.

#### 3.2.4. *tert*-Butyl (*R*)-2-fluoro-4-(5-(hydroxymethyl)-2-oxooxazolidin-3-yl)benzoate (**9**) [21]

To a solution of **8** (2.8 g, 8.0 mmol) in THF (80 mL), *n*-butyllithium in hexane (6 mL, 9.6 mmol) was added dropwise at −78 °C. After stirring for 1 h at −78 °C, (*R*)-glycidyl butyrate (1.2 mL, 8.8 mmol) was added, and the mixture was allowed to warm to room temperature. After stirring overnight at room temperature, saturated NH_4_Cl was added, and the organic materials were extracted with EtOAc. The organic layer was washed with brine, dried over anhydrous Na_2_SO_4_, filtered, and concentrated under reduced pressure. The resulting residue was separated by C.C. (hexane/EtOAc = 1:1) to yield **9** as a white solid (1.8 g, 71%). M.P. 143–145 °C. ^1^H-NMR (600 MHz, CDCl_3_) δ 7.87 (t, *J* = 8.4 Hz, 1H), 7.47 (dd, *J* = 13.1, 2.4 Hz, 1H), 7.26 (dd, *J* = 8.9, 2.4 Hz, 1H), 4.79–4.77 (m, 1H), 4.13–4.00 (m, 3H), 3.77 (d, *J* = 12.4 Hz, 1H), 1.59 (s, 9H), ^13^C-NMR (151 MHz, CDCl_3_) δ 163.0 (d, *J* = 4.3 Hz), 162.3 (d, *J* = 258.6 Hz), 154.3, 142.8 (d, *J* = 11.5 Hz), 132.8, 115.3 (d, *J* = 8.7 Hz), 112.4 (d, *J* = 2.9 Hz), 106.0 (d, *J* = 28.9 Hz), and 81.9, 62.5, 46.0, 28.2. HRMS (ESI): *m*/*z* calculated for C_15_H_18_FNNaO_5_: 334.1067, found [M + Na]^+^ 334.1076. ${\left[\alpha \right]}_{D}^{20}$ = −118.5° (c 0.10, CHCl_3_).

#### 3.2.5. *tert*-Butyl (*R*)-4-(5-(azidomethyl)-2-oxooxazolidin-3-yl)-2-fluorobenzoate (**10**) [21]

To a solution of **9** (2.5 g, 8.0 mmol) in DCM (80 mL), TEA (7 mL) and methanesulphonyl chloride (1.9 mL, 24 mmol) were added dropwise at 0 °C, and the mixture was allowed to warm to room temperature. After stirring for 1 h at room temperature, the solvent was removed by vacuum drying. The reaction mixture was diluted with water, and the organic material was extracted using EtOAc. The organic layer was dried over anhydrous Na_2_SO_4_, filtered, and concentrated under reduced pressure. The resulting residue (3.1 g, 8.0 mmol) was solved in DMF (35 mL), sodium azide (0.9 g, 15 mmol) was added. After stirring overnight at 60 °C, the mixture was diluted with hexane/EtOAc = 1:1. The organic layer was washed with brine, dried over anhydrous Na_2_SO_4_, and the filtrate was concentrated under reduced pressure. The resulting residue was separated by C.C. (hexane/EtOAc = 6:1) to yield **10** as a white solid (2.2 g, 82%). M.P. 93–95 °C. ^1^H-NMR (600 MHz, CDCl_3_) δ 7.87 (t, *J* = 8.4 Hz, 1H), 7.47 (dd, *J* = 13.1, 2.1 Hz, 1H), 7.26 (dd, *J* = 8.8, 2.2 Hz, 1H), 4.87–4.83 (m, 1H), 4.14–4.09 (m, 1H), 3.88 (dd, *J* = 8.9, 6.2 Hz, 1H), 3.76 (dd, *J* = 13.4, 4.1 Hz, 1H), 3.62 (dd, *J* = 13.4, 4.5 Hz, 1H), 1.59 (s, 9H), ^13^C-NMR (151 MHz, CDCl_3_) δ 162.9 (d, *J* = 2.9 Hz), 162.4 (d, *J* = 258.7 Hz), 153.5, 142.7 (d, *J* = 11.5 Hz), 132.8, 115.5 (d, *J* = 8.6 Hz), 112.4 (d, *J* = 2.9 Hz), 106.1 (d, *J* = 28.9 Hz), and 81.9, 70.9, 52.9, 47.0, 28.2. HRMS (ESI): *m*/*z* calculated for C_15_H_17_FN_4_NaO_4_: 359.1132, found [M + Na]^+^ 359.1133. ${\left[\alpha \right]}_{D}^{20}$ = −320.5° (c 0.20, CHCl_3_).

#### 3.2.6. *tert*-Butyl (*R*)-(4-(5-(azidomethyl)-2-oxooxazolidin-3-yl)-2-fluorophenyl)carbamate (**11**) [22]

To a solution of **10** (672 mg, 2.0 mmol) in DCM (4 mL) was added TFA (4 mL, 52 mmol) dropwise at 0 °C, and the mixture was allowed to warm to room temperature. After stirring overnight at room temperature, the solvent was removed in vacuo. To a solution of the resulting residue (565 mg, 2.0 mmol) in THF (7.5 mL), *tert*-butyl alcohol (*t*-BuOH) (950 μL, 10 mmol), TEA (360 μL, 2.6 mmol), and diphenylphosphoryl azide (DPPA) (560 μL, 2.6 mmol) were added. After stirring for 2 h at room temperature and overnight at 70 °C, the solvent was concentrated under reduced pressure, and the mixture was diluted with EtOAc. Saturated NaHCO_3_ was added to the mixture, and the organic materials were extracted with EtOAc. The organic layer was washed with brine, dried over anhydrous Na_2_SO_4_, filtered, and concentrated under reduced pressure. The resulting residue was separated by C.C. (hexane/EtOAc = 1:1) to yield **11** as a white solid (390 mg, 56%). M.P. 85–88 °C. ^1^H-NMR (600 MHz, CDCl_3_) δ 7.66–7.64 (m, 1H), 6.98 (d, *J* = 8.6 Hz, 1H), 6.67 (s, 1H), 4.79 (qd, *J* = 4.5, 1.7 Hz, 1H), 4.06 (t, *J* = 8.9 Hz, 1H), 3.83 (dd, *J* = 8.9, 6.2 Hz, 1H), 3.71 (dd, *J* = 13.4, 4.5 Hz, 1H), 3.59 (dd, *J* = 13.2, 4.6 Hz, 1H), 1.53 (s, 9H), ^13^C-NMR (151 MHz, CDCl_3_) δ 153.8, 152.4, 152.0 (d, *J* = 53.5 Hz), 133.1 (d, *J* = 10.1 Hz), 123.2 (d, *J* = 10.1 Hz), 113.3 (d, *J* = 2.8 Hz), 106.1 (d, *J* = 24.6 Hz),and 81.1, 70.6, 53.0, 47.4, 28.3. HRMS (ESI): *m*/*z* calculated for C_15_H_18_FN_5_NaO_4_: 374.1241, found [M + Na]^+^ 374.1241. [${\alpha ]}_{D}^{20}$ = −178.0° (c 0.20, CHCl_3_).

#### 3.2.7. *tert*-Butyl (*S*)-(4-(5-(aminomethyl)-2-oxooxazolidin-3-yl)-2-fluorophenyl)carbamate (**12**) [22]

To a solution of **11** (754 mg, 2.1 mmol) in methanol (MeOH) (50 mL), Pd/C (301 mg) was added. After stirring for 1 h under a H_2_ atmosphere at room temperature, the mixture was filtered through a Celite pad. The filtrate was concentrated under reduced pressure to obtain a colorless solid **12** (704 mg, quant.). M.P. 95–100 °C. ^1^H-NMR (600 MHz, CDCl_3_) δ 7.68 (d, *J* = 13.4 Hz, 1H), 6.99 (d, *J* = 8.9 Hz, 1H), 6.68 (bs, 1H), 4.68–4.66 (m, 1H), 4.02 (t, *J* = 8.8 Hz, 1H), 3.83 (t, *J* = 7.6 Hz, 1H), 3.11 (dd, *J* = 13.6, 4.0 Hz, 1H), 2.98 (dd, *J* = 13.6, 5.7 Hz, 1H), 1.53 (s, 9H), ^13^C-NMR (151 MHz, CDCl_3_) δ 154.5, 152.4, 152.0 (d, *J* = 243.0 Hz), 133.5 (d, *J* = 10.1 Hz), 129.7 (d, *J* = 24.6 Hz), 122.9 (d, *J* = 10.1 Hz), 113.2, 105.9 (d, *J* = 24.6 Hz), and 81.1, 73.8, 47.6, 44.9, 28.3. HRMS (ESI): *m*/*z* calculated for C_15_H_20_FN_3_NaO_4_: 348.1336, found [M + Na]^+^ 348.1329. ${\left[\alpha \right]}_{D}^{20}$ = −119.0° (c 0.20, CHCl_3_).

#### 3.2.8. *tert*-Butyl (*S*)-(4-(5-(acetamidomethyl)-2-oxooxazolidin-3-yl)-2-fluorophenyl)carbamate (**13**)

To a solution of **12** (978 mg, 3.0 mmol) in DCM (7 mL), TEA and Ac_2_O (473 μL, 4.3 mmol) were added dropwise at 0 °C, and the mixture was allowed to warm to room temperature. After stirring for 0.5 h at room temperature, the solvent was removed in vacuo, and a colorless solid **13** was obtained (814 mg, quant.). M.P. 105–108 °C. ^1^H-NMR (600 MHz, CDCl_3_) δ 7.90–8.17 (bs, 1H), 7.55 (dd, *J* = 13.1, 2.4 Hz, 1H), 6.99 (dd, *J* = 8.9, 1.4 Hz, 1H), 6.69 (bs, 1H), 6.59 (t, *J* = 6.2 Hz, 1H), 4.80–4.76 (m, 1H), 4.02 (t, *J* = 8.9 Hz, 1H), 3.76 (dd, *J* = 9.1, 6.7 Hz, 1H), 3.68–3.63 (m, 2H), 2.03 (s, 3H), 1.53 (s, 9H), ^13^C-NMR (151 MHz, CDCl_3_) δ171.4, 154.4, 152.5, 152.0 (d, *J* = 242.8 Hz), 133.1 (d, *J* = 10.5 Hz), 123.2 (d, *J* = 11.5 Hz), 113.5 (d, *J* = 2.8 Hz), 106.0 (d, *J* = 2.6 Hz), and 81.2, 72.0, 47.5, 41.9, 28.3, 23.0. HRMS (ESI): *m*/*z* calculated for C_17_H_22_FN_3_NaO_5_: 390.1441, found [M + Na]^+^ 390.1432. ${\left[\alpha \right]}_{D}^{20}$ = −102.5° (c 0.20, CHCl_3_).

#### 3.2.9. (*S*)-*N*-((3-(4-Amino-3-fluorophenyl)-2-oxooxazolidin-5-yl)methyl)acetamide (**14**)

To a solution of **13** (332 mg, 0.9 mmol) in DCM (1 mL), TFA (1 mL) was added. After stirring for 1 h at room temperature, the solvent was removed in vacuo, and a colorless solid **14** was obtained (215 mg, 90%). M.P. 92–95 °C. ^1^H-NMR (600 MHz, CD_3_OD) δ 7.58 (dd, *J* = 12.7, 2.4 Hz, 1H), 7.49 (t, *J* = 8.6 Hz, 1H), 7.23–7.21 (m, 1H), 4.73–4.69 (m, 1H), 4.06 (t, *J* = 8.9 Hz, 1H), 3.73 (dd, *J* = 9.1, 6.4 Hz, 1H), 3.47–3.46 (m, 2H), 1.86 (s, 3H), ^13^C-NMR (151 MHz, CD_3_OD) δ 172.8, 156.7, 156.1 (d, *J* = 37.5 Hz), 155.0 (d, *J* = 13.0 Hz), 138.5 (d, *J* = 10.1 Hz), 126.6, 118.3 (d, *J* = 13.0 Hz), 113.4 (d, *J* = 2.9 Hz), 106.0 (d, *J* = 24.6 Hz), 72.2, 41.8, 21.1.

#### 3.2.10. (*S*)-*N*-((3-(3-Fluoro-4-((2-nitrophenyl)sulfonamido)phenyl)-2-oxooxazolidin-5-yl)methyl)acetamide (**5**)

To a solution of **14** (59 mg, 0.2 mmol) in pyridine (Py) (1.5 mL), 2-nitrobenzenesulfonyl (Ns) chloride was added. After stirring for 1.5 h at room temperature, the solvent was concentrated under reduced pressure, and the mixture was diluted with CHCl_3_. Saturated NH_4_Cl was added to the mixture, and the organic materials were extracted with CHCl_3_. The organic layer was washed with brine, dried over anhydrous Na_2_SO_4_, filtered, and concentrated under reduced pressure. The residue was separated by C.C. (CHCl_3_/MeOH = 40:1) to yield **5** as a yellow solid (91 mg, 92%). M.P. 98–100 °C. ^1^H-NMR (600 MHz, CDCl_3_) δ 7.92 (dd, *J* = 7.9, 1.0 Hz, 1H), 7.81 (dd, *J* = 7.9, 1.4 Hz, 1H), 7.75 (td, *J* = 7.7, 1.4 Hz, 1H), 7.63 (td, *J* = 7.7, 1.1 Hz, 1H), 7.54 (t, *J* = 8.6 Hz, 1H), 7.46 (dd, *J* = 12.4, 2.4 Hz, 1H), 7.10 (dt, *J* = 8.9, 1.4 Hz, 1H), 6.42 (t, *J* = 6.2 Hz, 1H), 4.80–4.78 (m, 1H), 4.03 (t, *J* = 9.1 Hz, 1H), 3.77 (dd, *J* = 9.1, 6.7 Hz, 1H), 3.68–3.63 (m, 2H), 2.01 (s, 3H), ^13^C-NMR (151 MHz, CDCl_3_) δ171.4, 155.8 (d, *J* = 247.1 Hz), 154.2, 147.9, 137.7 (d, *J* = 10.1 Hz), 134.3, 132.8, 132.6, 131.2, 127.5, 125.7, 119.0 (d, *J* = 14.5 Hz), 113.6, 113.6, 106.1 (d, *J* = 26.0 Hz), and 72.1, 47.4, 41.8, 23.1. HRMS (ESI): *m*/*z* calculated for C_18_H_17_FN_4_NaO_7_S: 475.0700, found [M + Na]^+^ 475.0705. ${\left[\alpha \right]}_{D}^{20}$ = −70.5° (c 0.20, CHCl_3_).

#### 3.2.11. (*S*)-*N*-((3-(4-((*N*-(2-((tert-Butyldimethylsilyl)oxy)ethyl)-2-nitrophenyl)sulfonamido)-3-fluorophenyl)-2-oxooxazolidin-5-yl)methyl)acetamide (**15**)

To a solution of **5** (113 mg, 0.25 mmol) in DMF (0.3 mL), (2-bromoethoxy)-*tert*-butyldimethylsilane (107 μL, 0.5 mmol) and K_2_CO_3_ (52 mg, 0.75 mmol) were added. After stirring for 2 h at room temperature and 48 h at 50 °C, the reaction mixture was diluted with water, and the organic material was extracted with hexane/EtOAc (1:1). The organic layer was dried over anhydrous Na_2_SO_4_, filtered, and concentrated under reduced pressure. The residue was separated by C.C. (CHCl_3_/MeOH = 40:1) to yield **15** as a yellow solid (137 mg, 86%). M.P. 88–90 °C. ^1^H-NMR (600 MHz, CDCl_3_) δ 6.80–6.77 (m, 2H), 6.70 (dd, *J* = 7.7, 0.9 Hz, 1H), 6.67–6.60 (m, 2H), 6.53 (t, *J* = 8.8 Hz, 1H), 6.22 (dd, *J* = 8.6, 2.1 Hz, 1H), 5.28 (t, *J* = 6.2 Hz, 1H), 3.90–3.88 (m, 1H), 3.15 (t, *J* = 9.1 Hz, 1H), 2.94–2.87 (m, 3H), 2.82–2.78 (m, 3H), 2.74–2.71 (m, 1H), 1.13 (s, 3H), -0.08 (s, 9H), ^13^C-NMR (151 MHz, CDCl_3_) δ 171.1,159.7 (d, *J* = 251.4 Hz), 154.0, 148.0, 139.7 (d, *J* = 10.2 Hz), 134.1, 133.7, 132.4, 131.3, 131.0, 124.0, 121.0 (d, *J* = 13.0 Hz), 113.1, 106.5 (d, *J* = 26.0 Hz), and 72.0, 61.1, 53.4, 47.4, 41.9, 25.8, 23.1, 18.1, -5.5. HRMS (ESI): *m*/*z* calculated for C_26_H_35_FN_4_NaO_8_SSi: 633.1827, found [M + Na]^+^ 633.1805. ${\left[\alpha \right]}_{D}^{20}$ = −87.5° (c 0.20, CHCl_3_).

#### 3.2.12. (*S*)-*N*-((3-(3-Fluoro-4-((*N*-(2-hydroxyethyl)-2-nitrophenyl)sulfonamido)phenyl)-2-oxooxazolidin-5-yl)methyl)acetamide (**16**)

To a solution of **15** (137 mg, 0.23 mmol) in THF (0.5 mL), Tetrabutylammonium fluoride (TBAF) (350 μL, 0.35 mmol) was added dropwise at 0 °C, and the mixture was allowed to warm to room temperature. After stirring for 2 h at room temperature, the solvent was removed in vacuo. The reaction mixture was diluted with water, and the organic material was extracted with EtOAc. The organic layer was dried over anhydrous Na_2_SO_4_, filtered, and concentrated under reduced pressure. The residue was separated by C.C. (CHCl_3_/MeOH = 10:1) to yield **16** as a yellow solid (99 mg, 85%). M.P. 102–104 °C. ^1^H-NMR (600 MHz, CD_3_OD) δ 7.80 (ddd, *J* = 8.3, 7.0, 1.1 Hz, 1H), 7.74 (dd, *J* = 7.9, 1.4 Hz, 1H), 7.70 (dd, *J* = 7.9, 1.4 Hz, 1H), 7.66 (td, *J* = 7.6, 1.1 Hz, 1H), 7.56 (dd, *J* = 13.1, 2.4 Hz, 1H), 7.43 (t, *J* = 8.6 Hz, 1H), 7.31–7.29 (m, 1H), 4.81–4.80 (m, 1H), 4.15 (t, *J* = 8.9 Hz, 1H), 3.82 (dd, *J* = 9.3, 6.2 Hz, 3H), 3.59–3.56 (m, 4H), 1.96 (s, 3H), ^13^C-NMR (151 MHz, CD_3_OD) δ 174.1, 161.2 (d, *J* = 250.0 Hz), 156.2, 149.6, 142.1 (d, *J* = 11.5 Hz), 135.6, 135.0, 133.0, 132.6, 132.2, 125.3, 121.6 (d, *J* = 13.0 Hz), 114.8, 107.5 (d, *J* = 27.4 Hz), and 73.6, 60.8, 54.5, 49.9, 49.5, 49.3, 49.2, 49.0, 48.9, 48.8, 48.6, 43.1, 22.5. HRMS (ESI): *m*/*z* calculated for C_20_H_22_FN_4_O_8_S: 497.1142, found [M + H]^+^ 497.1154. ${\left[\alpha \right]}_{D}^{20}$ = −64.5° (c 0.20, CH_3_OH).

#### 3.2.13. *tert*-Butyl (*S*)-2-(2-((*N*-(4-(5-(acetamidomethyl)-2-oxooxazolidin-3-yl)-2-fluorophenyl)-2-nitrophenyl)sulfonamido)ethoxy)acetate (**17**)

To a solution of **16** (30 mg, 0.06 mmol) in DCM (1 mL), *tert*-butyl bromoacetate (13 μL, 0.09 mmol), tetrabutylammonium hydrogen sulfate (20 mg, 0.06 mmol), and 1 M NaOH (90 μL, 0.09mmol) were added. After stirring vigorously for 3 h at room temperature, 1 M NaOH (90 μL, 0.09 mmol) was added to the reaction mixture. After stirring vigorously for 1 h at room temperature, the reaction mixture was diluted with water, and the organic material was extracted with DCM. The organic layer was dried over Na_2_SO_4_, filtered, and concentrated under reduced pressure. The residue was separated by HPLC (CHCl_3_/MeOH = 40:1) to yield **17** as a yellow solid (16 mg, 75% based on conversion yield, 43% recovered **16**). ^1^H-NMR (600 MHz, CDCl_3_) δ 7.73–7.68 (m, 2H), 7.61 (dd, *J* = 7.9, 1.4 Hz, 1H), 7.58–7.52 (m, 2H), 7.47 (t, *J* = 8.6 Hz, 1H), 7.13 (dd, *J* = 8.9, 2.4 Hz, 1H), 6.17 (t, *J* = 6.0 Hz, 1H), 4.79 (q, *J* = 3.0 Hz, 1H), 4.06 (t, *J* = 8.9 Hz, 1H), 3.92 (s, 2H), 3.78 (dd, *J* = 9.3, 6.9 Hz, 1H), 3.73–3.66 (m, 3H), 3.64–3.60 (m, 1H), 2.04 (s, 3H), 1.46 (s, 9H), ^13^C-NMR (151 MHz, CDCl_3_) δ 172.4, 160.8 (d, *J* = 248.7 Hz), 151.0 (d, *J* = 11.6 Hz), 147.9, 137.8, 133.7, 133.1, 132.3, 131.5, 131.4, 123.8, 123.2, 112.1 (d, *J* = 11.5 Hz), 109.9, 108.9, 99.5 (d, *J* = 24.6 Hz), and 69.1, 59.9, 59.0, 54.0, 46.5, 43.7, 29.7, 24.0, 23.1, 19.7, 13.7. HRMS (ESI): *m*/*z* calculated for C_26_H_31_FN_4_NaO_10_S: 633.1643, found [M + Na]^+^ 633.1651. ${\left[\alpha \right]}_{D}^{20}$ = −77.0° (c 0.20, CHCl_3_).

#### 3.2.14. (*S*)-2-(2-((4-(5-(Acetamidomethyl)-2-oxooxazolidin-3-yl)-2-fluorophenyl)amino)ethoxy)acetic acid (**1**)

To a solution of **17** (30 mg, 0.06 mmol) in DMF (300 μL), K_2_CO_3_ (30 mg, 0.22 mmol) and thiophenol (17 μL, 0.17 mmol) were added. After stirring for 6 h at room temperature, the reaction mixture was diluted with water, and the organic material was extracted with DCM. The organic layer was dried over Na_2_SO_4_, filtered, and concentrated under reduced pressure. The resulting residue was separated by HPLC (CHCl_3_/MeOH = 40:1) to yield a colorless solid (16 mg, 86%). To a solution of the solid (15 mg, 0.04 mmol) in DCM (440 μL), TFA was added. After stirring for 1 h at room temperature and concentrating under reduced pressure, a colorless solid was obtained. To a solution of the solid in water, 1 M NaOH (60 μL) was added dropwise. The crude solution was purified using Sep Pak C18 (H_2_O-MeOH) to yield PNU-142300 (**1**) as a sodium salt (19 mg, 94%). M.P. 101–103 °C. ^1^H-NMR (600 MHz, CD_3_OD) δ 7.34 (dd, *J* = 13.4, 2.4 Hz, 1H), 7.03 (dt, *J* = 8.8, 1.3 Hz, 1H), 6.79 (t, *J* = 9.1 Hz, 1H), 4.75 (dd, *J* = 8.9, 6.2 Hz, 1H), 4.08 (t, *J* = 8.9 Hz, 1H), 3.89 (s, 2H), 3.75 (dd, *J* = 9.1, 6.4 Hz, 1H), 3.69 (t, *J* = 5.5 Hz, 2H), 3.54 (d, *J* = 4.8 Hz, 2H), 3.33 (t, *J* = 5.3 Hz, 2H), 1.97 (s, 3H), ^13^C-NMR (151 MHz, CD_3_OD) δ 178.1, 174.1, 157.1, 153.4 (d, *J* = 238.4 Hz), 135.6 (d, *J* = 11.5 Hz), 129.0, 116.9, 113.5 (d, *J* = 4.3 Hz), and 108.5, 108.4, 73.4, 71.7, 70.3, 49.8, 44.5, 43.2, 22.5. HRMS (ESI): *m*/*z* calculated for C_16_H_20_FN_3_NaO_6_: 392.1234, found [M + H]^+^ 392.1219. ${\left[\alpha \right]}_{D}^{20}$ = −63.0° (c 0.20, CH_3_OH).

#### 3.2.15. (*S*)-*N*-((3-(4-((*N*-(2-(Benzyloxy)ethyl)-2-nitrophenyl)sulfonamido)-3-fluorophenyl)-2-oxooxazolidin-5-yl)methyl)acetamide (**18**)

To a solution of **5** (45 mg, 0.1 mmol) in DMF (0.5 mL), K_2_CO_3_ (21 mg, 0.15 mmol) and benzyl 2-bromoethyl ether (32 μL, 0.2 mmol) were added. After stirring overnight at 50 °C, the reaction mixture was diluted with water, and the organic material was extracted with DCM. The organic layer was dried over Na_2_SO_4_, filtered, and concentrated under reduced pressure. The resulting residue was separated by C.C. (CHCl_3_/MeOH = 30:1) to yield **18** as a colorless solid (137 mg, 93%). ^1^H-NMR (600 MHz, CDCl_3_) δ 7.72 (dd, *J* = 8.1, 1.2 Hz, 1H), 7.65 (td, *J* = 7.7, 1.4 Hz, 1H), 7.58 (dd, *J* = 8.1, 1.2 Hz, 1H), 7.51–7.48 (m, 2H), 7.35–7.25 (m, 4H), 7.22 (d, *J* = 6.5 Hz, 2H), 7.08 (dd, *J* = 8.9, 2.4 Hz, 1H), 6.42 (t, *J* = 6.2 Hz, 1H), 4.79–4.75 (m, 1H), 4.43 (s, 2H), 4.02–3.96 (m, 2H), 3.75 (dd, *J* = 9.1, 6.7 Hz, 1H), 3.67 (ddd, *J* = 14.6, 6.0, 3.6 Hz, 1H), 3.62–3.56 (m, 3H), 2.01 (s, 3H), ^13^C-NMR (151 MHz, CDCl_3_) δ 171.3, 159.8 (d, *J* = 252.9 Hz), 154.0, 148.0, 139.8 (d, *J* = 10.1 Hz), 137.8, 133.8 (d, *J* = 8.6 Hz), 132.3, 131.4, 131.1, 128.3, 127.7, 127.7, 123.9, 120.5 (d, *J* = 11.5 Hz), 113.3, 106.4 (d, *J* = 26.0 Hz), and 72.8, 72.0, 67.9, 50.9, 47.4, 41.8, 23.1. HRMS (ESI): *m*/*z* calculated for C_27_H_27_FN_4_NaO_8_S: 609.1431, found [M + Na]^+^ 609.1418. ${\left[\alpha \right]}_{D}^{20}$ = −85.5° (c 0.20, CHCl_3_).

#### 3.2.16. (*S*)-*N*-((3-(4-((2-(Benzyloxy)ethyl)amino)-3-fluorophenyl)-2-oxooxazolidin-5-yl)methyl)acetamide (**19**)

To a solution of **18** (79 mg, 0.14 mmol) in DMF (0.5 mL), K_2_CO_3_ (75 mg, 0.54 mmol), and thiophenol (41 μL, 0.4 mmol) were added. After stirring for 6 h at room temperature, the reaction mixture was diluted with water, and the organic material was extracted with DCM. The organic layer was dried over Na_2_SO_4_, filtered, and concentrated under reduced pressure. The resulting residue was separated by HPLC (CHCl_3_/MeOH = 40:1) to yield a colorless solid **19** (15.6 mg, 86%). M.P. 80–83 °C. ^1^H-NMR (600 MHz, CDCl_3_) δ 7.37–7.30 (m, 6H), 6.97 (dq, J = 8.7, 1.2 Hz, 1H), 6.66 (t, *J* = 9.1 Hz, 1H), 6.23 (bs, 1H), 4.74 (t, *J* = 3.1 Hz, 1H), 4.56 (s, 2H), 4.19–4.31 (1H), 3.99 (t, *J* = 9.1 Hz, 1H), 3.72–3.68 (m, 4H), 3.61–3.57 (m, 1H), 3.33 (t, *J* = 4.6 Hz, 2H), 2.02 (s, 3H), ^13^C-NMR (151 MHz, CDCl_3_) δ13C-NMR (151 MHz, CHLOROFORM-D) δ 171.1, 154.6, 151.3 (d, *J* = 240.0 Hz), 137.9, 133.9 (d, *J* = 11.5 Hz), 128.5, 127.8, 127.8, 114.9, 112.2 (d, *J* = 4.4 Hz), 107.0 (d, *J* = 23.1 Hz), and 73.2, 71.8, 68.3, 48.0, 43.5, 42.0, 23.1. HRMS (ESI): *m*/*z* calculated for C_21_H_24_FN_3_NaO_4_: 424.1649, found [M + Na]^+^ 424.1671. ${\left[\alpha \right]}_{D}^{20}$ = −89.5° (c 0.20, CHCl_3_).

#### 3.2.17. Ethyl (*S*)-*N*-(4-(5-(Acetamidomethyl)-2-oxooxazolidin-3-yl)-2-fluorophenyl)-*N*-(2-(benzyloxy)ethyl)glycinate (**20**)

To a solution of **19** (20 mg, 0.05 mmol) in acetonitrile (MeCN) (0.5 mL), K_2_CO_3_ (11 mg, 0.08 mmol) and ethyl bromoacetate (16 μL, 0.1 mmol) were added. After stirring for 2 days at 90 °C, the reaction mixture was diluted with water, and the organic material was extracted with DCM. The organic layer was dried over Na_2_SO_4_, filtered, and concentrated under reduced pressure. The resulting residue was separated by HPLC (CHCl_3_/MeOH = 40:1) to yield **20** a colorless solid (16 mg, 68%). ^1^H-NMR (600 MHz, CDCl_3_) δ 7.42–7.22 (m, 6H), 7.03–6.99 (m, 1H), 6.93 (t, *J* = 9.3 Hz, 1H), 6.44–6.38 (m, 1H), 4.76–4.69 (m, 1H), 4.49 (s, 2H), 4.16–4.07 (m, 3H), 3.99 (t, *J* = 9.1 Hz, 1H), 3.75–3.66 (m, 4H), 3.61–3.54 (m, 3H), 3.48 (s, 2H), 2.06–1.96 (m, 3H), 1.22 (t, *J* = 7.2 Hz, 3H), ^13^C-NMR (151 MHz, CDCl_3_) δ 171.2, 171.1, 154.4, 154.3 (d, *J* = 244.2 Hz), 138.2, 134.3 (d, *J* = 8.6 Hz), 131.4 (d, *J* = 10.1 Hz), 128.5, 128.4, 127.8, 127.6, 127.5, 120.1 (d, *J* = 4,2 Hz), 114.0, 107.8 (d, *J* = 26.0 Hz), 73.2, 71.9, 69.1, 60.7, 54.4 (d, *J* = 4.4 Hz), 52.5, 50.8, 47.7, 41.9, 23.1, 14.2. HRMS (ESI): *m*/*z* calculated for C_25_H_30_FN_3_NaO_6_: 510.2016, found [M + Na]^+^ 510.2015. ${\left[\alpha \right]}_{D}^{20}$ = −86.0° (c 0.20, CHCl_3_).

#### 3.2.18. Ethyl (*S*)-*N*-(4-(5-(Acetamidomethyl)-2-oxooxazolidin-3-yl)-2-fluorophenyl)-*N*-(2-hydroxyethyl)glycinate (**21**)

To a solution of **20** (16 mg, 0.04 mmol) in THF (1 mL), Pd/C (20 mg) was added. After stirring for 24 h under a H_2_ atmosphere at room temperature, the mixture was filtered through a Celite pad. The filtrate was concentrated under reduced pressure, and a colorless solid **21** was obtained (20 mg, quant.). M.P. 75–78 °C. ^1^H-NMR (600 MHz, CDCl_3_) δ 7.40–7.37 (m, 1H), 7.02–7.00 (m, 2H), 6.77–6.77 (m, 1H), 4.76–4.75 (m, 1H), 4.20 (q, *J* = 7.1 Hz, 1H), 4.01–3.97 (m, 3H), 3.75–3.72 (m, 2H), 3.67–3.61 (m, 4H), 3.47 (t, *J* = 4.6 Hz, 2H), 2.01 (s, 3H), 1.27 (q, *J* = 7.1 Hz, 3H), ^13^C-NMR (151 MHz, CDCl_3_) δ 172.6, 171.4, 155.0 (d, *J* = 244.2 Hz), 154.5, 133.6 (d, *J* = 10.1 Hz), 132.3 (d, *J* = 10.1 Hz), 120.9 (d, *J* = 2.9 Hz), 114.0, 107.8 (d, *J* = 26.0 Hz), and 72.0, 61.3, 59.5, 55.5, 55.5, 47.6, 41.9, 23.0, 14.2. ${\left[\alpha \right]}_{D}^{20}$ = −55.0° (c 0.20, CHCl_3_).

#### 3.2.19. (*S*)-*N*-(4-(5-(Acetamidomethyl)-2-oxooxazolidin-3-yl)-2-fluorophenyl)-*N*-(2-hydroxyethyl)glycine (**2**)

To a solution of **21** (40 mg, 0.01 mmol) in THF (1 mL), 1 M NaOH (300 μL) was added. After stirring for 2 h at room temperature, the supernatant was removed to obtain a colorless solid. A solution of the solid in water was purified by Sep Pak C18 (water, MeOH) to yield PNU-142586 (**2**) as a sodium salt (38 mg, 80%). M.P. 80–82 °C.^1^H-NMR (600 MHz, CD_3_OD) δ 7.37 (dd, *J* = 15.8, 2.7 Hz, 1H), 7.06–7.04 (m, 1H), 6.93 (t, *J* = 9.5 Hz, 1H), 4.75 (dd, *J* = 8.8, 6.4 Hz, 1H), 4.09 (t, *J* = 9.1 Hz, 1H), 3.79 (d, *J* = 1.4 Hz, 2H), 3.75 (dd, *J* = 9.3, 6.5 Hz, 1H), 3.66 (t, *J* = 5.2 Hz, 2H), 3.54–3.50 (m, 4H), 1.96 (s, 3H), ^13^C-NMR (151 MHz, CD_3_OD) δ180.2, 174.1, 156.9, 154.8 (d, *J* = 242.7 Hz), 136.1, 131.7 (d, *J* = 10.1 Hz), and 120.1, 115.9, 109.2 (d, *J* = 27.5 Hz), 73.4, 60.9, 59.1, 56.9, 43.2, 22.5. HRMS (ESI): *m*/*z* calculated for C_16_H_20_FN_3_NaO_6_: 392.1234, found [M + H]^+^ 392.1252. ${\left[\alpha \right]}_{D}^{20}$ = −52.0° (c 0.20, CH_3_OH).

#### 3.2.20. (*S*)-*N*-((3-(3-Fluoro-4-((2-hydroxyethyl)amino)phenyl)-2-oxooxazolidin-5-yl)methyl)acetamide (**3**)

To a solution of **19** (15 mg, 0.04 mmol) in THF (1 mL), Pd/C (13 mg) was added. After stirring overnight under a H_2_ atmosphere at room temperature, the mixture was filtered through a Celite pad. The filtrate was concentrated under reduced pressure, and a colorless solid **3** was obtained (9 mg, 86%). M.P. 98–100 °C. ^1^H-NMR (600 MHz, CD_3_OD) δ 7.35 (dd, *J* = 13.6, 2.6 Hz, 1H), 7.03 (dq, *J* = 8.8, 1.2 Hz, 1H), 6.78 (t, *J* = 9.3 Hz, 1H), 4.76–4.74 (m, 1H), 4.07 (t, *J* = 8.9 Hz, 1H), 3.76–3.72 (m, 3H), 3.54 (d, *J* = 4.8 Hz, 2H), 3.26 (t, *J* = 5.7 Hz, 2H), 1.96 (s, 3H), ^13^C-NMR (151 MHz, CD_3_OD) δ 174.1, 157.1, 152.5 (d, *J* = 238.4 Hz), 135.5 (d, *J* = 13.0 Hz), 129.0 (d, *J* = 10.1 Hz), 116.9, 113.3 (d, *J* = 4.4 Hz), 108.4 (d, *J* = 24.6 Hz), and 73.4, 61.4, 49.7, 49.4, 49.3, 49.1, 49.0, 48.9, 48.7, 48.6, 46.7, 43.2, 30.9, 22.4. HRMS (ESI): *m*/*z* calculated for C_14_H_18_FN_3_NaO_4_: 334.1179, found [M + Na]^+^ 334.1263. ${\left[\alpha \right]}_{D}^{20}$ = −48.5° (c 0.20, CH_3_OH).

#### 3.2.21. Ethyl (*S*)-*N*-(4-(5-(Acetamidomethyl)-2-oxooxazolidin-3-yl)-2-fluorophenyl)-*N*-((4-nitrophenyl)sulfonyl)glycinate (**22**)

To a solution of **5** (100 mg, 0.22 mmol) in DMF (0.2 mL), K_2_CO_3_ (45 mg, 0.33 mmol), and ethyl bromoacetate (49 μL) were added. After stirring overnight at 50 °C, the reaction mixture was diluted with water, and the organic material was extracted with hexane/EtOAc (1:1). The organic layer was dried over Na_2_SO_4_, filtered, and concentrated under reduced pressure. The resulting residue was separated by C.C. (CHCl_3_/MeOH = 20:1) to yield **22** as a colorless solid (112 mg, 95%). M.P. 78–80 °C. ^1^H-NMR (600 MHz, CDCl_3_) δ 7.73–7.66 (m, 4H), 7.60–7.53 (m, 2H), 7.12 (dd, *J* = 8.6, 2.1 Hz, 1H), 6.37 (s, 1H), 4.80–4.79 (m, 1H), 4.52 (s, 2H), 4.16 (q, *J* = 7.1 Hz, 2H), 4.06 (t, *J* = 8.9 Hz, 1H), 3.80 (dd, *J* = 9.1, 6.7 Hz, 1H), 3.68–3.61 (m, 2H), 2.02 (s, 3H), 1.25 (t, *J* = 7.0 Hz, 3H), ^13^C-NMR (151 MHz, CDCl_3_) δ 171.3, 168.7, 159.4 (d, *J* = 251.4 Hz), 154.0, 147.8, 140.2 (d, *J* = 10.1 Hz), 134.4, 134.1, 132.4, 131.7, 131.3, 124.3, 120.8 (d, *J* = 13.0 Hz), 113.3, 106.4 (d, *J* = 26.0 Hz), and 72.0, 61.7, 52.5, 47.4, 41.9, 23.1, 14.1. HRMS (ESI): *m*/*z* calculated for C_22_H_23_FN_4_NaO_9_S: 561.1067, found [M + Na]^+^ 561.1073. ${\left[\alpha \right]}_{D}^{20}$ = −138.0° (c 0.20, CHCl_3_).

#### 3.2.22. Ethyl (*S*)-(4-(5-(Acetamidomethyl)-2-oxooxazolidin-3-yl)-2-fluorophenyl)glycinate (**23**)

To a solution of **22** (81 mg, 0.15 mmol) in DMF (0.2 mL), K_2_CO_3_ (83 mg, 0.6 mmol) and thiophenol (46 μL, 0.45 mmol) were added. After stirring overnight at room temperature, the reaction mixture was diluted with water, and the organic material was extracted with hexane/EtOAc (1:1). The organic layer was dried over Na_2_SO_4_, filtered, and concentrated under reduced pressure. The resulting residue was separated by HPLC (CHCl_3_/MeOH = 1:1) to yield **23** a colorless solid (50 mg, 95%). M.P. 93–95 °C. ^1^H-NMR (600 MHz, CDCl_3_) δ 7.36 (dd, *J* = 13.1, 2.4 Hz, 1H), 6.96 (dq, *J* = 8.7, 1.2 Hz, 1H), 6.88 (t, *J* = 6.0 Hz, 1H), 6.54 (t, *J* = 8.9 Hz, 1H), 4.76–4.73 (m, 1H), 4.24 (q, *J* = 7.1 Hz, 2H), 3.98 (t, *J* = 8.9 Hz, 1H), 3.92 (s, 2H), 3.73–3.69 (m, 2H), 3.65–3.58 (m, 2H), 2.01 (s, 3H), 1.30 (t, *J* = 7.2 Hz, 3H), ^13^C-NMR (151 MHz, CDCl_3_) δ 171.5, 170.7, 154.8, 151.1 (d, *J* = 239.8 Hz), 132.7 (d, *J* = 11.6 Hz), 128.5 (d, *J* = 8.6 Hz), 114.9 (d, *J* = 2.9 Hz), 112.1 (d, *J* = 4.4 Hz), 107.1 (d, *J* = 23.2 Hz), and 72.0, 61.5, 48.0, 45.5, 41.9, 23.0, 14.2. HRMS (ESI): *m*/*z* calculated for C_16_H_20_FN_3_NaO_5_: 376.1285, found [M + Na]^+^ 376.1277. ${\left[\alpha \right]}_{D}^{20}$ = −36.0° (c 0.20, CHCl_3_).

#### 3.2.23. (*S*)-(4-(5-(Acetamidomethyl)-2-oxooxazolidin-3-yl)-2-fluorophenyl)glycine (**4**)

To a solution of **23** (18 mg, 0.05 mmol) in THF (1 mL), 1M NaOH (79 μL) was added. After stirring for 2 h at room temperature, MeOH was added, and the reaction mixture was subjected to PTLC (CHCl_3_/MeOH = 1:1) to yield PNU-173558 (**4**) as a sodium salt (15 mg, 95%). M.P. 118–120 °C. ^1^H-NMR (600 MHz, CD_3_OD) δ 7.36 (dd, *J* = 13.4, 2.4 Hz, 1H), 7.01 (dt, *J* = 8.7, 1.3 Hz, 1H), 6.64 (t, *J* = 9.3 Hz, 1H), 4.76–4.74 (m, 1H), 4.08 (t, *J* = 9.1 Hz, 1H), 3.75 (dd, *J* = 9.1, 6.4 Hz, 1H), 3.65 (s, 2H), 3.54 (d, *J* = 5.2 Hz, 2H), 1.97 (s, 3H), ^13^C-NMR (151 MHz, CD_3_OD) δ 177.6, 174.0, 170.4, 157.1, 153.3 (d, *J* = 236.9 Hz), 135.5 (d, *J* = 13.0 Hz), 128.7 (d, *J* = 10.1 Hz), 116.9, 113.1 (d, *J* = 4.4 Hz), 108.3 (d, *J* = 24.4 Hz), and 73.4, 49.8, 48.4, 43.2, 30.8, 22.4. HRMS (ESI): *m*/*z* calculated for C_14_H_16_FN_3_NaO_5_: 348.0972, found [M + H]^+^ 348.0986. ${\left[\alpha \right]}_{D}^{20}$ = −69.5° (c 0.20, CH_3_OH).

## 4. Conclusions

In conclusion, we presented a novel synthesis route for linezolid metabolites using the Ns strategy. Four linezolid metabolites were synthesized from a common intermediate (**5**), which is Ns-protected aniline derivative obtained by *Curtius* rearrangement reaction of oxazolidinone derivatives. The protection of aromatic amines by the Ns group was effective in preparing the desired secondary and tertiary amine derivatives. This is the first report on the synthesis of PNU-142618 and PNU-173558. The linezolid metabolites obtained in this study are currently being used in clinical analysis of the pharmacokinetics of these metabolites, and the results will be reported in due course. The results in this study not only contribute to the pharmacokinetic and toxicological studies of linezolid metabolites but also suggest that the morpholine ring of linezolid can be replaced with various other substituents, and it is expected that novel linezolid derivatives with antimicrobial activity will be developed.

## Data Availability

The original contributions presented in this study are included in the article/Supplementary Materials. Further inquiries can be directed to the corresponding authors.

## Supplementary Materials

The following supporting information can be downloaded at: https://www.mdpi.com/article/10.3390/ph18121821/s1, NMR spectrum of the synthesized derivatives (compound **1**–**23**).

## Abbreviations

The following abbreviations are used in this manuscript:

C.C.	Column chromatography
DCM	Dichloromethane
DMF	*N*,*N*-dimethylformamide
HPLC	High-performance liquid chromatography
TBAF	Tetrabutylammonium fluoride
TEA	Triethylamine
TFA	Trifluoroacetic acid
THF	Tetrahydrofuran
