# Supplementary material for: A Unified Approach to Synthesizing Four Linezolid Metabolites That May Cause Thrombocytopenia
Source: Pharmaceuticals (Basel). 2025 Nov 28;18(12):1821. doi: 10.3390/ph18121821 (PMC12735765; doi:10.3390/ph18121821)
Supplement: Supplementary file 1 [file pharmaceuticals-18-01821-s001.zip › pharmaceuticals-3984896-supplementary.pdf]

*Supplementary Materials*

# A Unified Approach to Synthesizing Four Linezolid Metabolites that May Cause Thrombocytopenia

**Naoki Oikawa [1,*], Natsu Inoue [1], Shogo Ishii [1], Takumi Goto [1], Hiroaki Saito [1], Fumihiro Kurosaki [2], Takahiro Aoyama [2], Yasuhiro Tsuji [2] and Taketo Uchiyama [1,*]**

[1] Laboratory of Medicinal Chemistry, School of Pharmacy, Nihon University, 7-7-1, Narashinodai, Funabashi Chiba, 274-8555, Japan; phna17028@g.nihon-u.ac.jp; shogo.i.1120@gmail.com; g.takumi.0315@gmail.com; saito.hiroaki@nihon-u.ac.jp
[2] Laboratory of Clinical Pharmacometrics, School of Pharmacy, Nihon University, 7-7-1, Narashinodai, Funabashi Chiba, 274-8555, Japan; kurosaki.fumihiro@nihon-u.ac.jp; aoyama.takahiko@nihon-u.ac.jp; tsuji.yasuhiro@nihon-u.ac.jp

***** Correspondence: oikawa.naoki@nihon-u.ac.jp; uchiyama.taketo@nihon-u.ac.jp

## NMR spectrum of the synthesized derivatives

*tert*-Butyl 2-fluoro-4-nitrobenzoate (**6**)

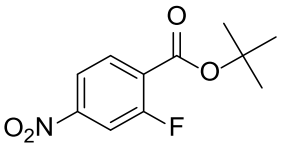


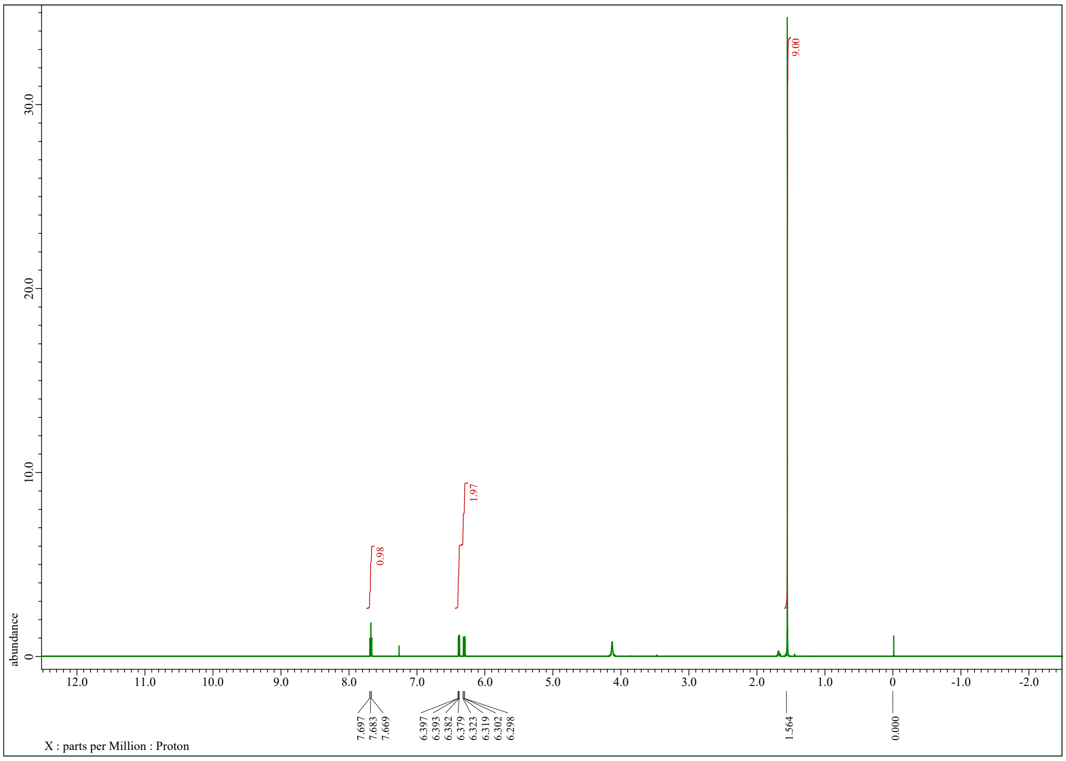


Figure S1. $^1$H-NMR spectrum of compound **6**

*tert*-Butyl 4-amino-2-fluorobenzoate (**7**)

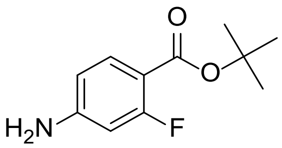

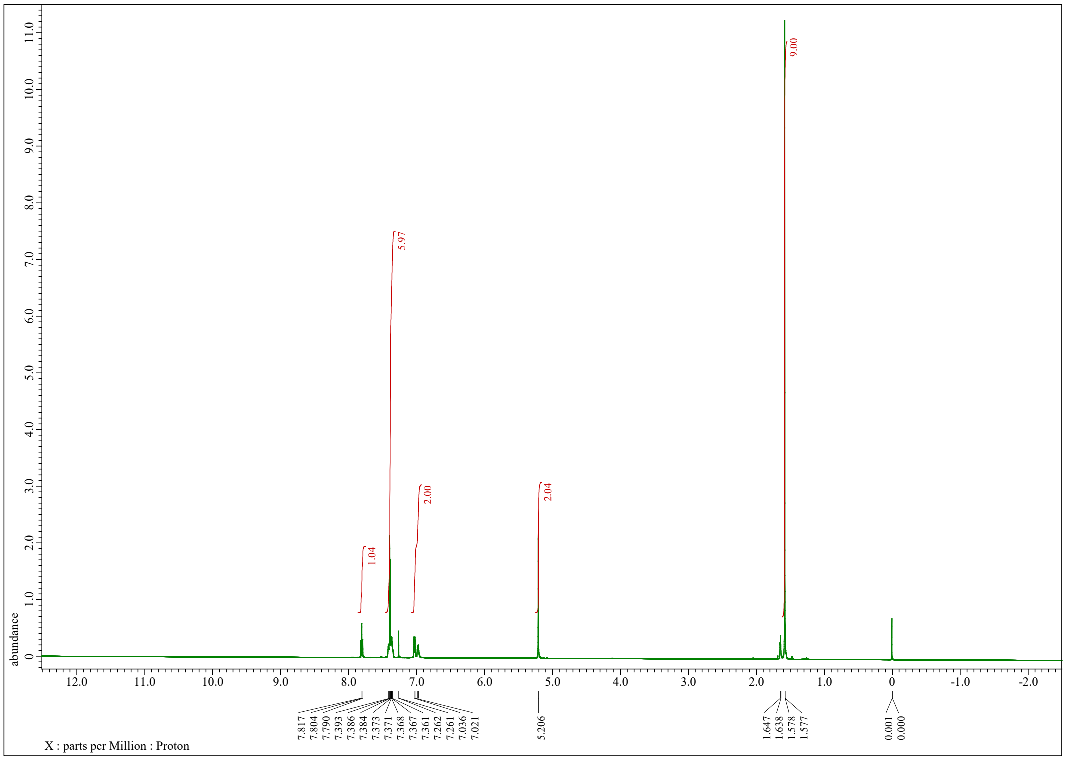


Figure S2. $^{1}$H-NMR spectrum of compound **7**

*tert*-Butyl 4-(((benzyloxy)carbonyl)amino)-2-fluorobenzoate (**8**)

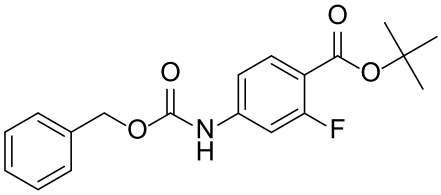

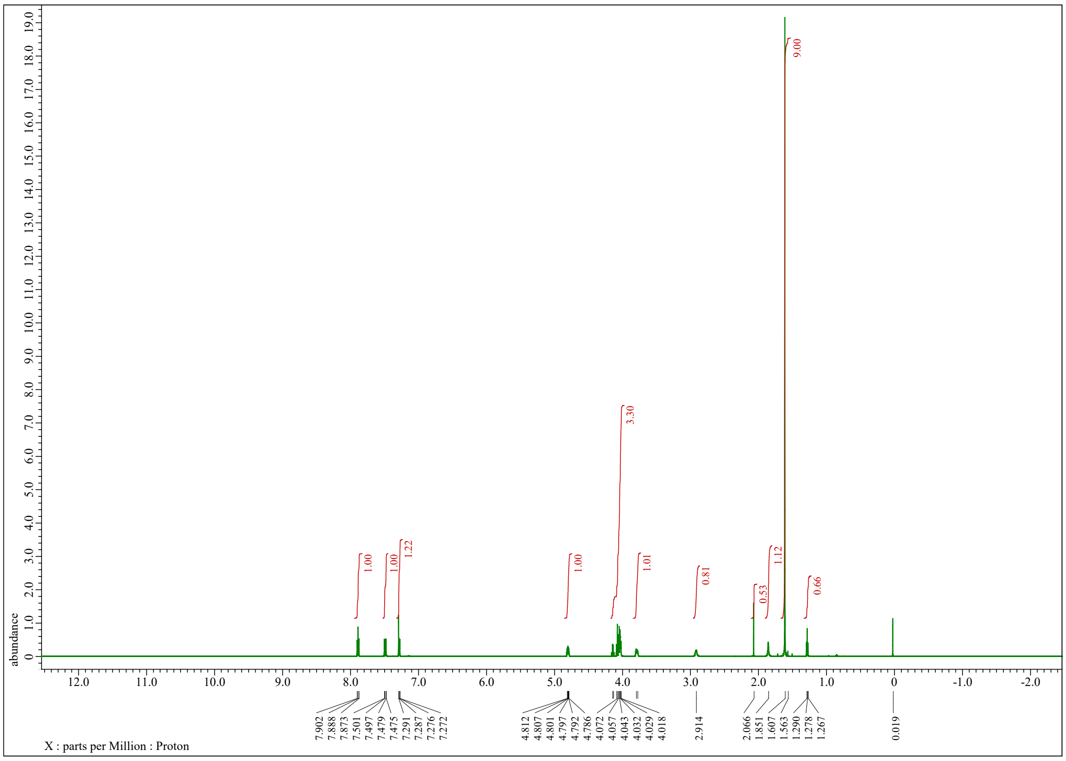

Figure S3. $^{1}$H-NMR spectrum of compound **8**

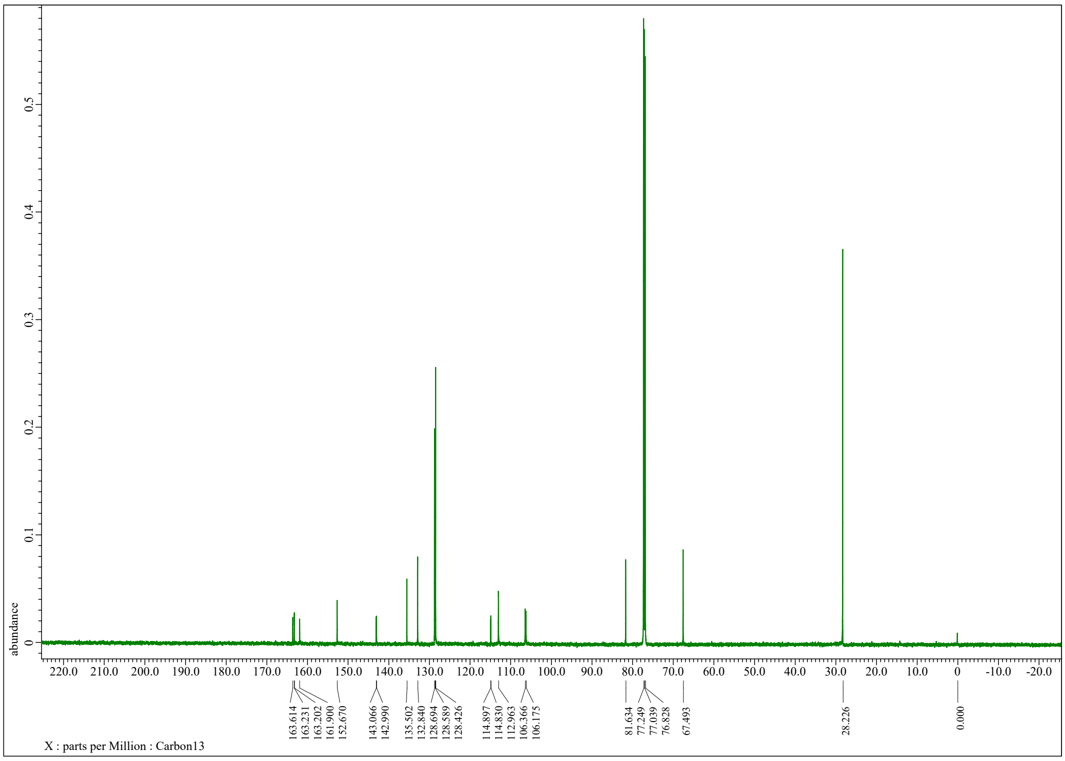

Figure S4. $^{13}$C-NMR spectrum of compound **8**

*tert*-Butyl (R)-2-fluoro-4-(5-(hydroxymethyl)-2-oxooxazolidin-3-yl)benzoate (**9**)

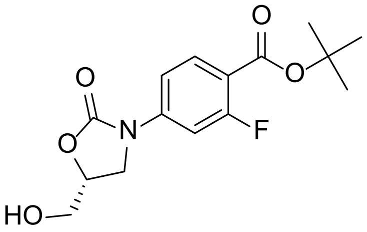

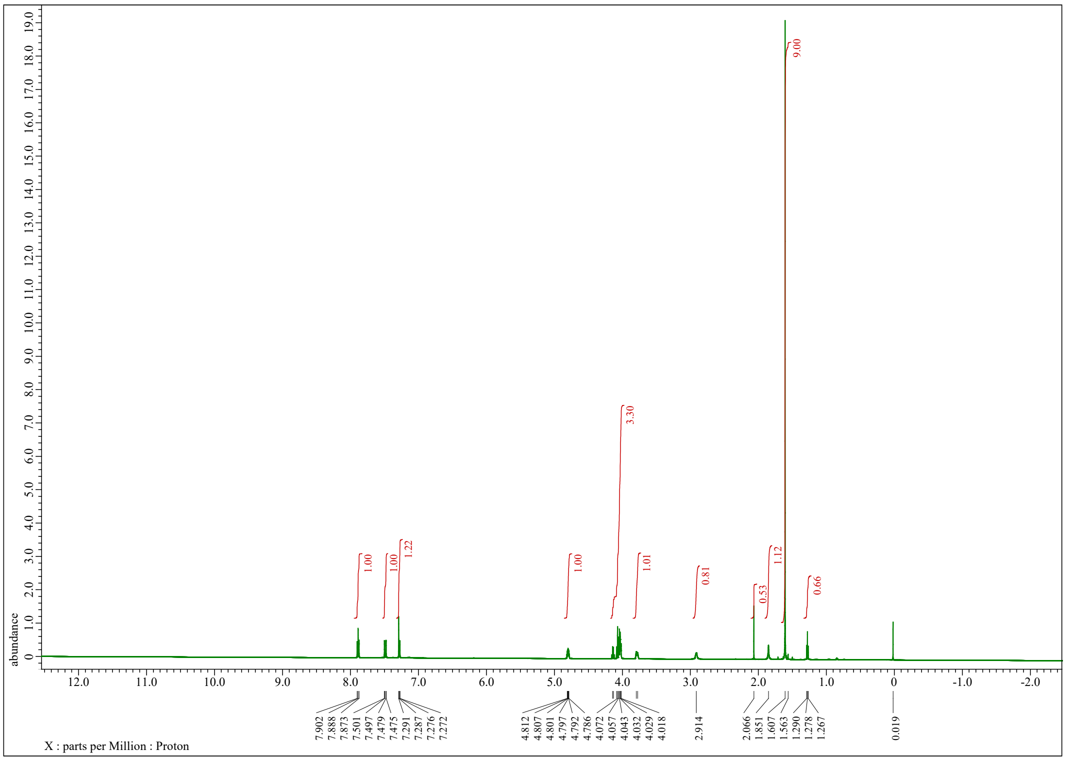


Figure S5. $^{1}$H-NMR spectrum of compound **9**

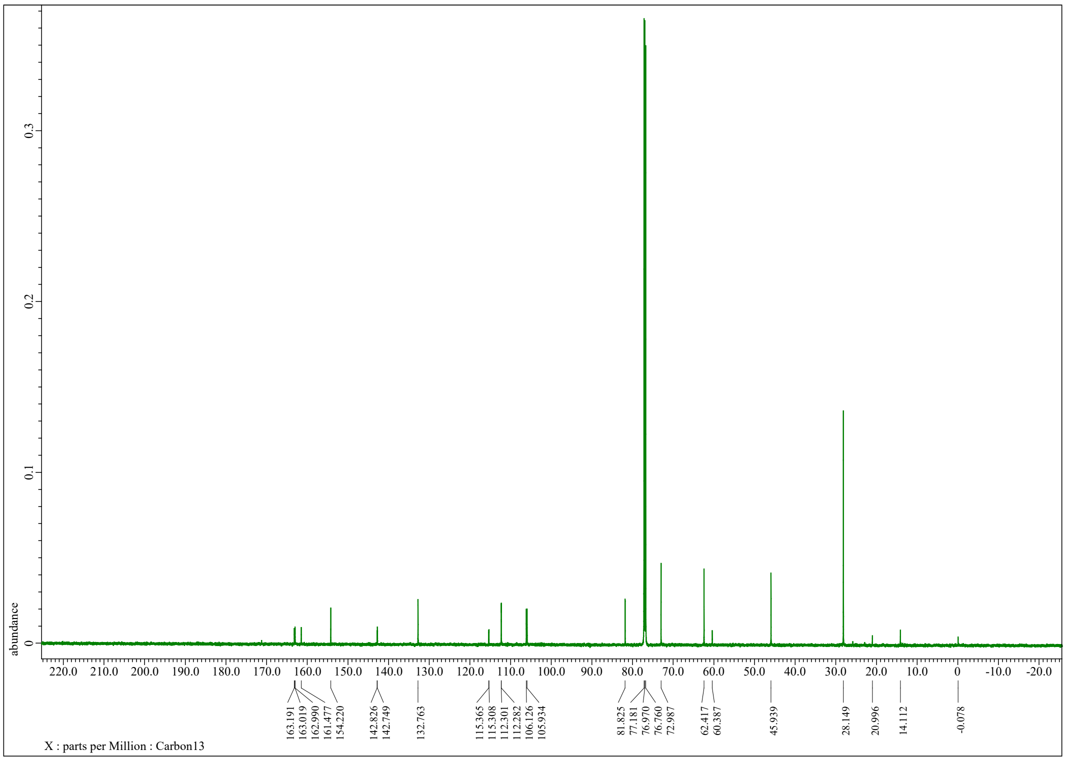


Figure S6. $^{13}$C-NMR spectrum of compound **9**

*tert*-Butyl (R)-4-(5-(azidomethyl)-2-oxooxazolidin-3-yl)-2-fluorobenzoate (**10**)

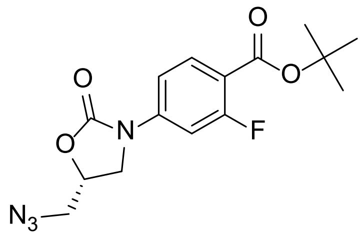

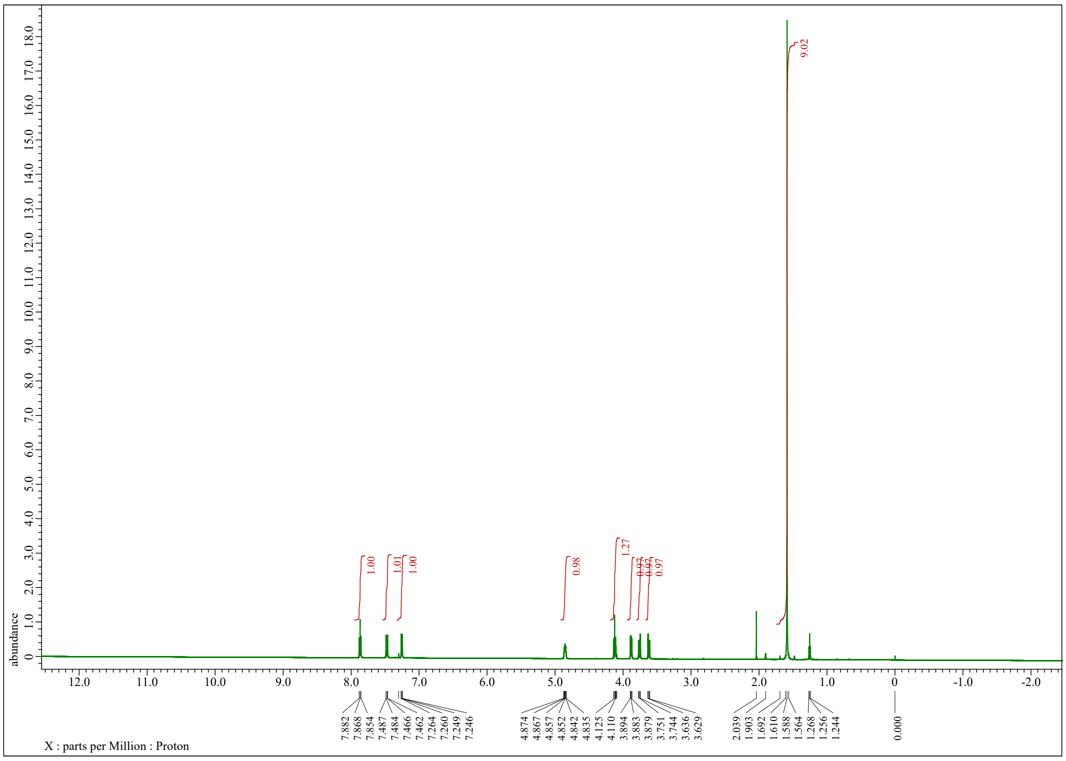

Figure S7. $^{1}$H-NMR spectrum of compound **10**

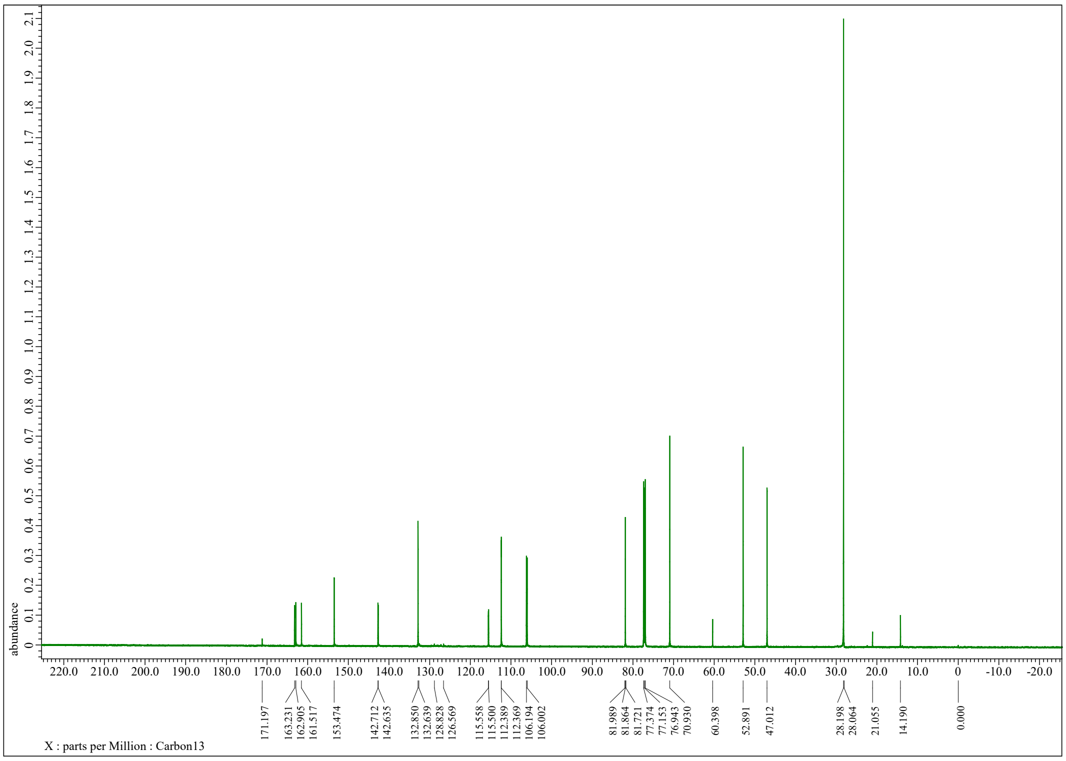

Figure S8. $^{13}$C-NMR spectrum of compound **10**

*tert*-Butyl (*R*)-(4-(5-(azidomethyl)-2-oxooxazolidin-3-yl)-2-fluorophenyl)carbamate (**11**)

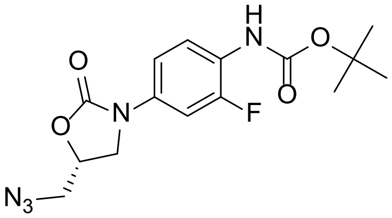

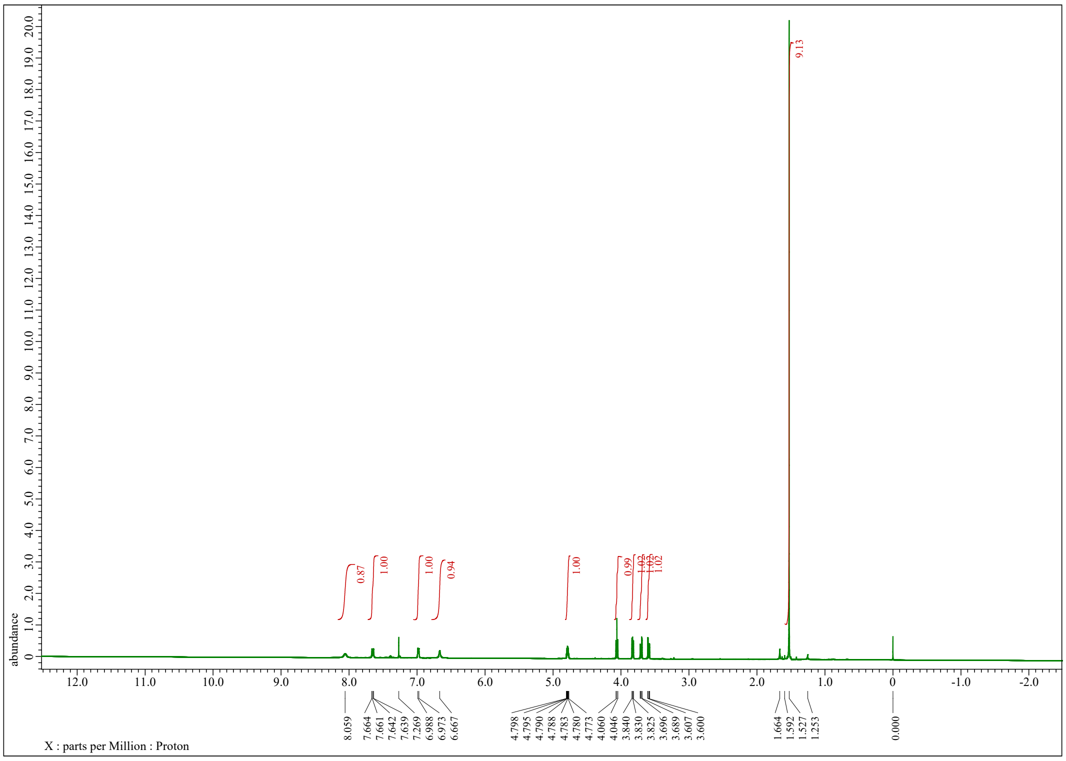

Figure S9. $^1$H-NMR spectrum of compound **11**

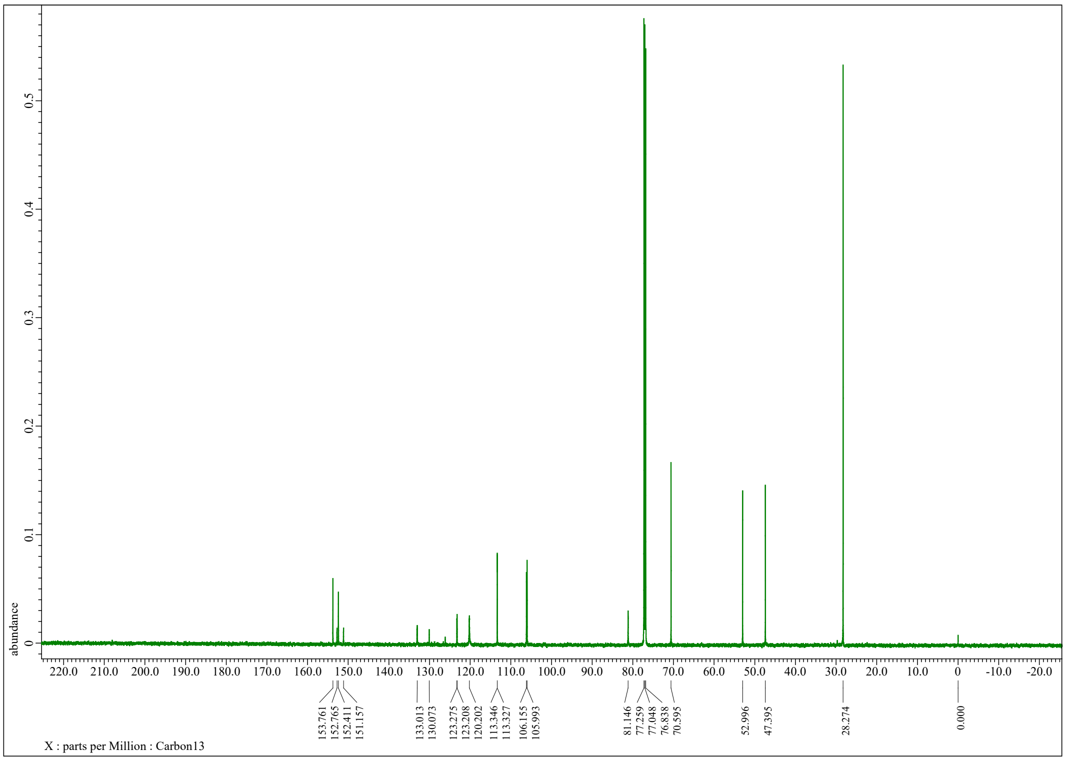

Figure S10. $^{13}$C-NMR spectrum of compound **11**

*tert*-Butyl (*S*)-(4-(5-(aminomethyl)-2-oxooxazolidin-3-yl)-2-fluorophenyl)carbamate (**12**)

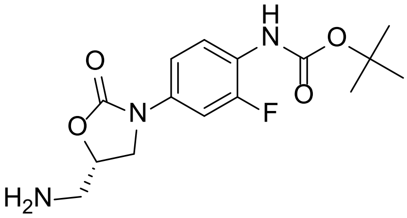

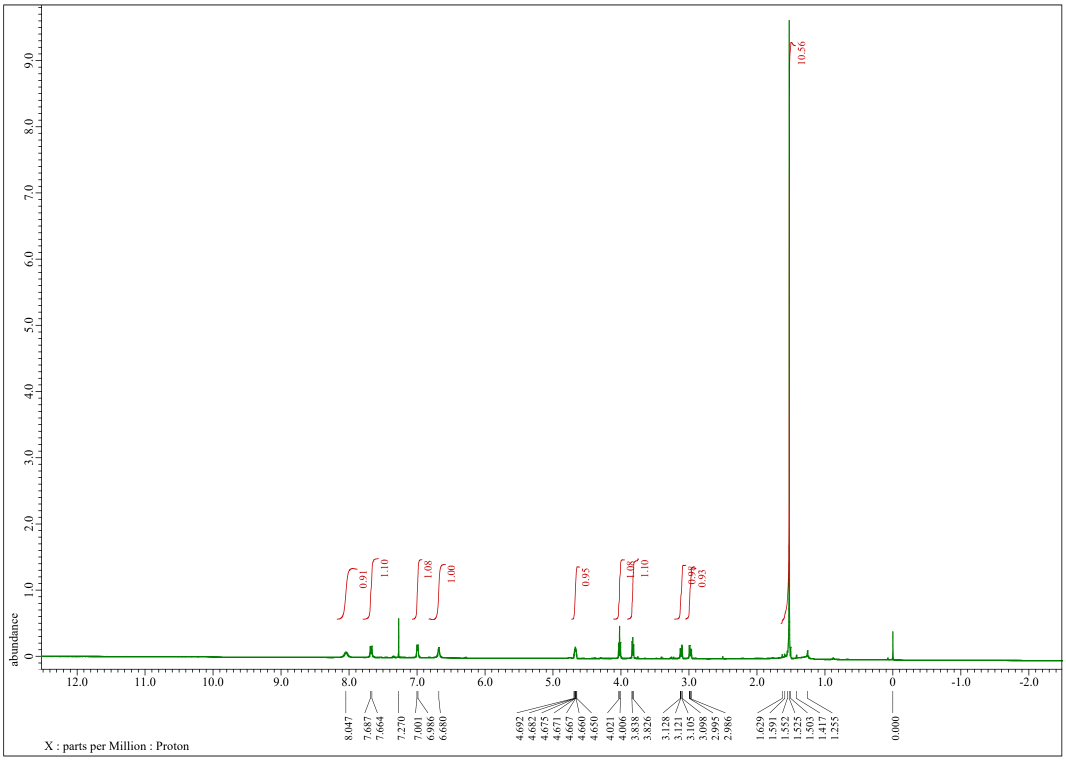


Figure S11. $^{1}$H-NMR spectrum of compound **12**

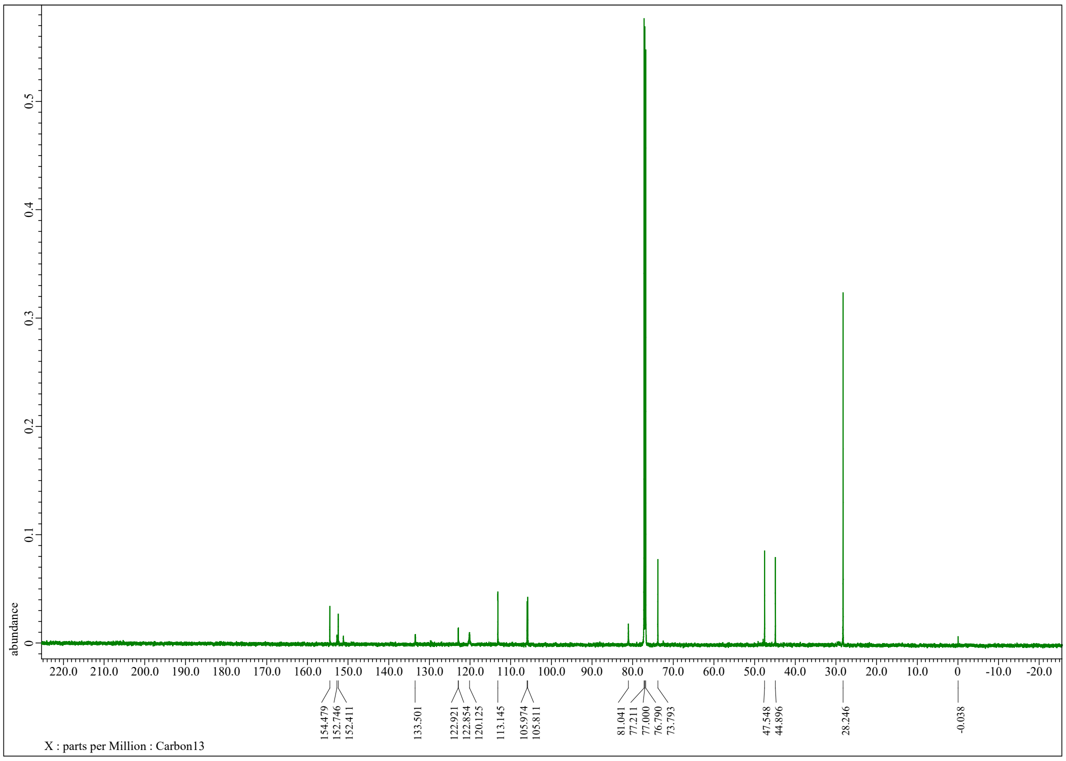


Figure S12. $^{13}$C-NMR spectrum of compound **12**

*tert*-Butyl (*S*)-(4-(5-(acetamidomethyl)-2-oxooxazolidin-3-yl)-2-fluorophenyl)carbamate (**13**)

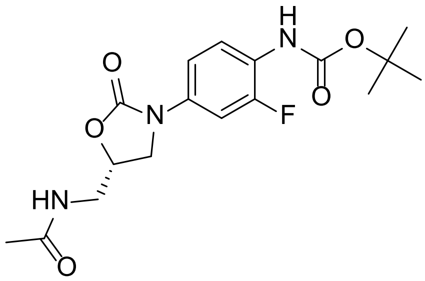

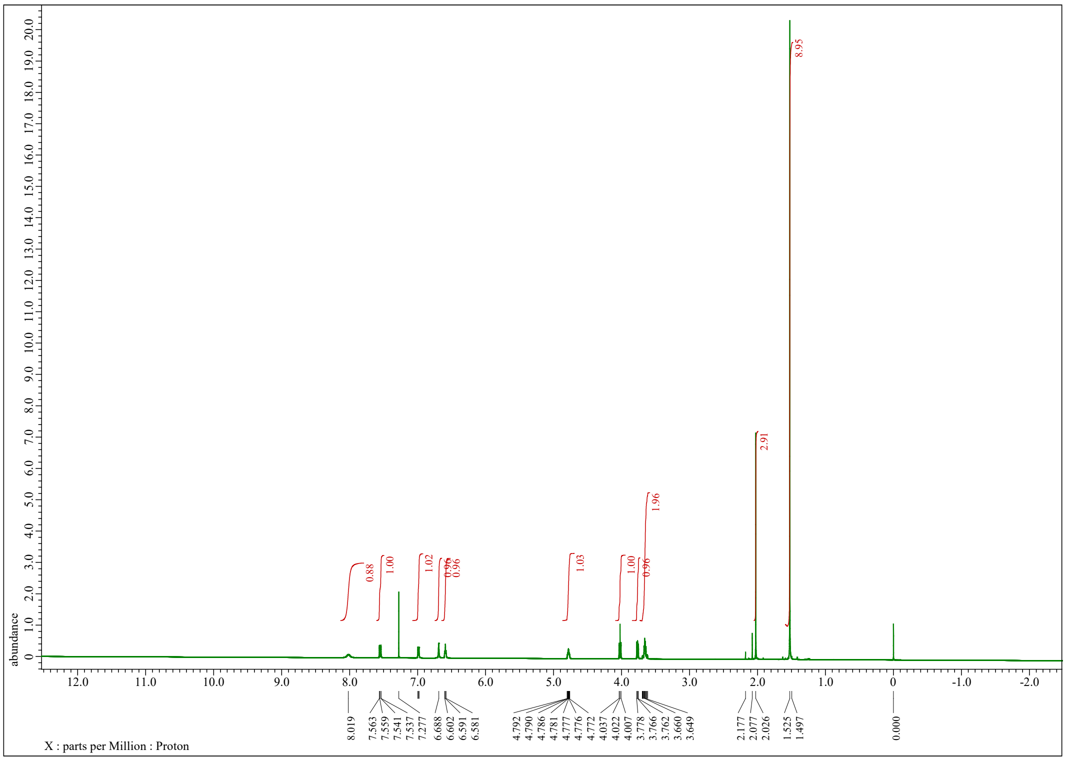

Figure S13. $^{1}$H-NMR spectrum of compound **13**

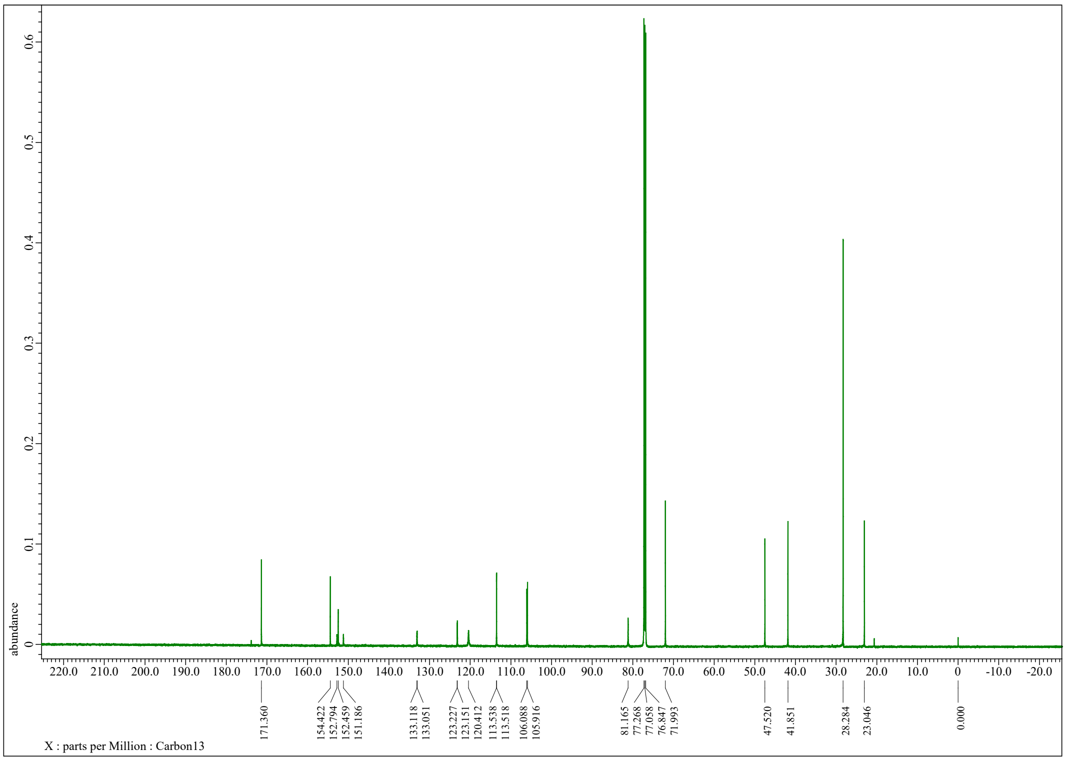

Figure S14. $^{13}$C-NMR spectrum of compound **13**

(*S*)-*N*-((3-(4-Amino-3-fluorophenyl)-2-oxooxazolidin-5-yl)methyl)acetamide (**14**)

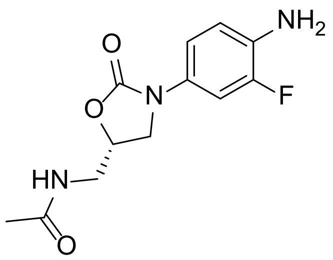


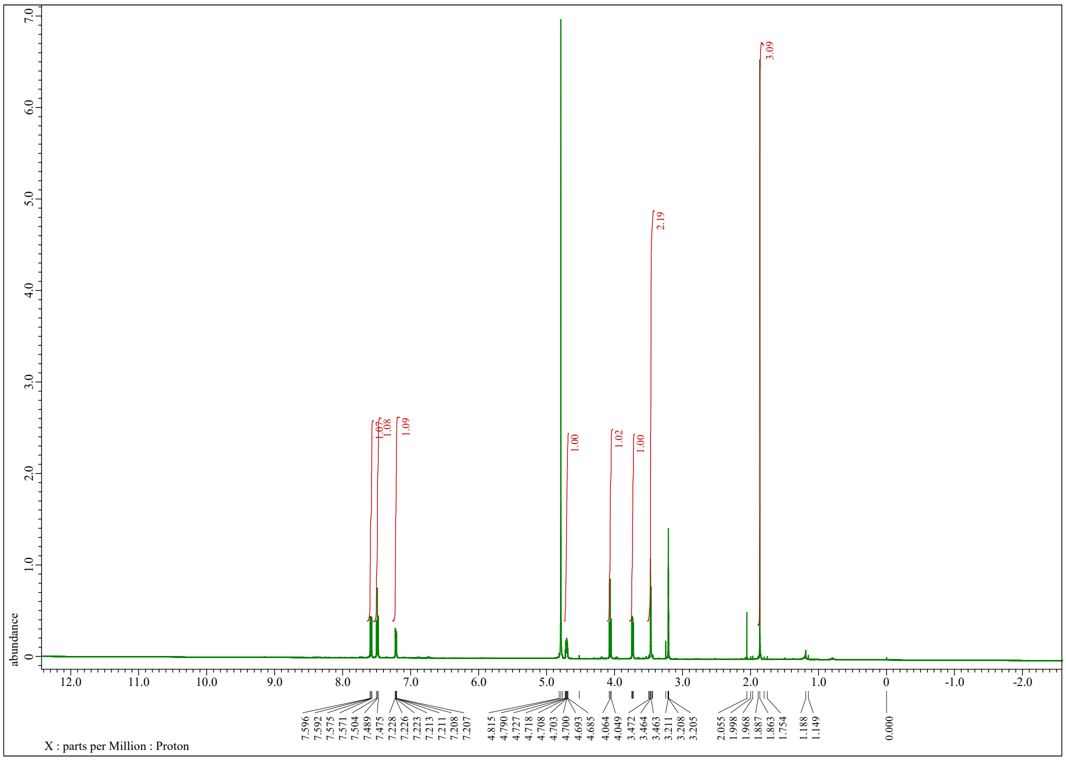


Figure S15. $^{1}$H-NMR spectrum of compound **14**

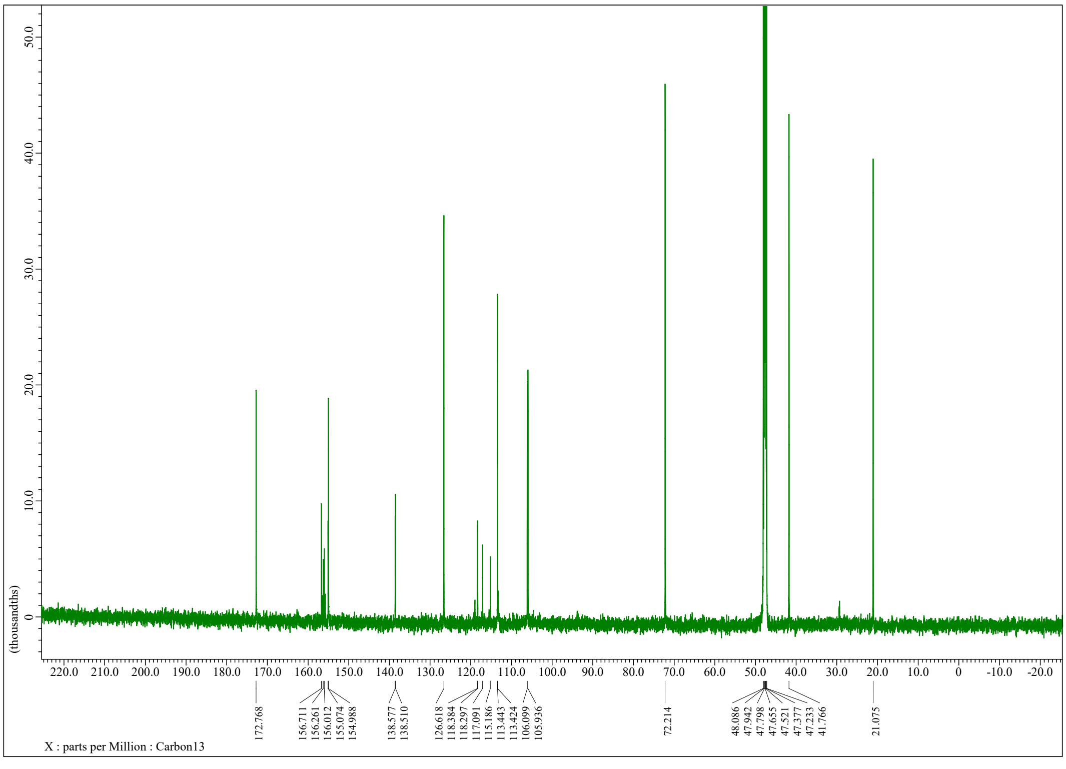


Figure S16. $^{13}$C-NMR spectrum of compound **14**

(*S*)-*N*-((3-(3-Fluoro-4-((2-nitrophenyl)sulfonamido)phenyl)-2-oxooxazolidin-5-yl)methyl)acetamide (**5**)

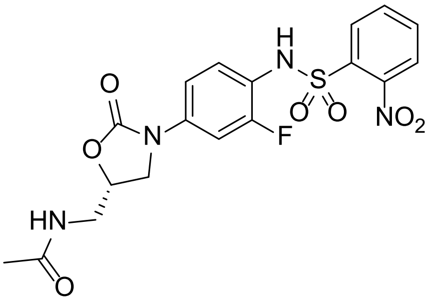


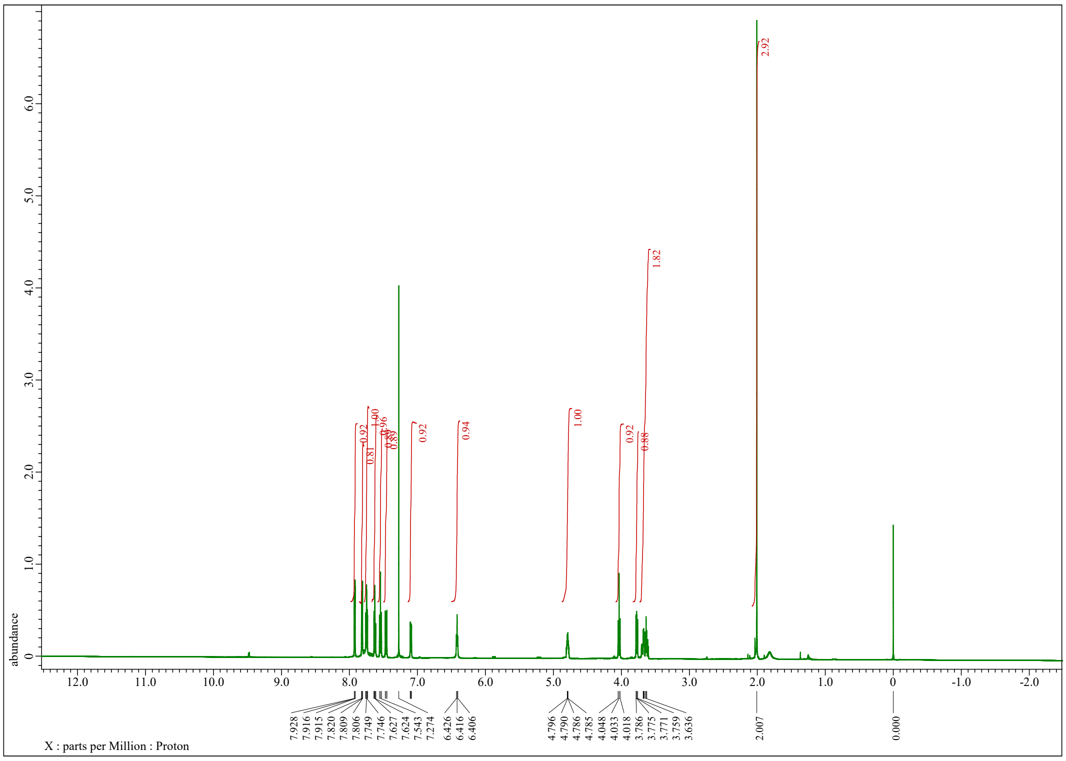


Figure S17. $^{1}$H-NMR spectrum of compound **5**

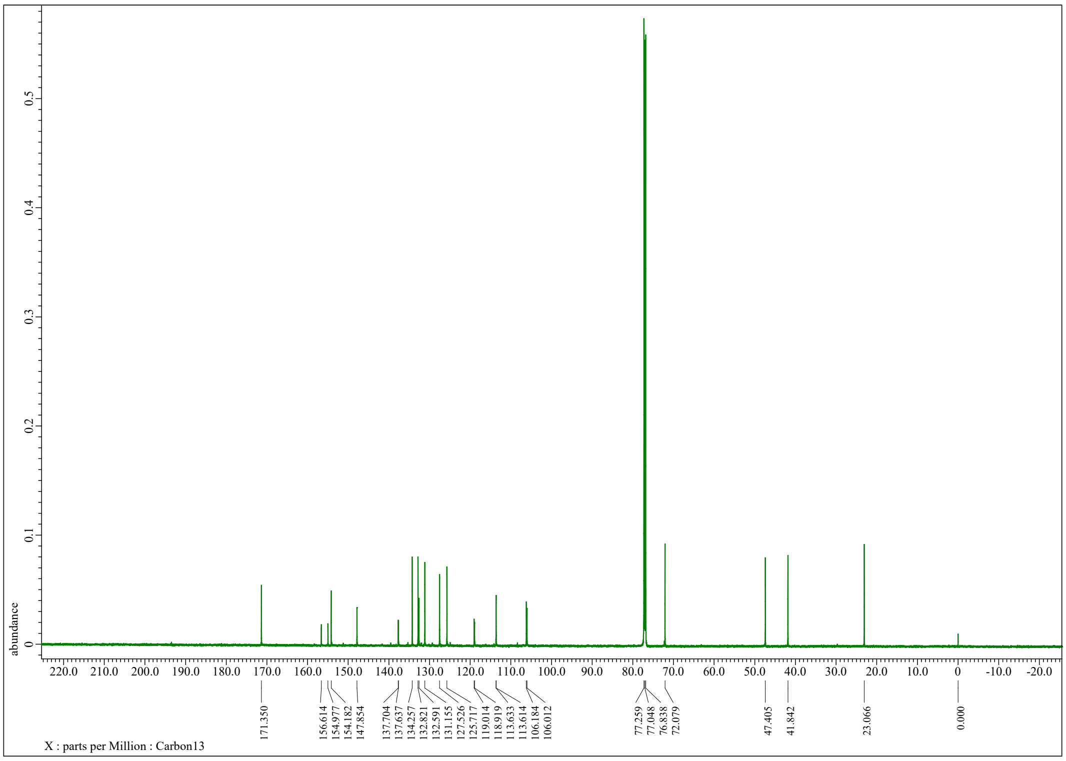


Figure S18. $^{13}$C-NMR spectrum of compound **5**

(*S*)-*N*-((3-(4-((*N*-(2-((*tert*-Butyldimethylsilyl)oxy)ethyl)-2-nitrophenyl)sulfonamido)-3-fluorophenyl)-2-oxooxazolidin-5-yl)methyl)acetamide (**15**)

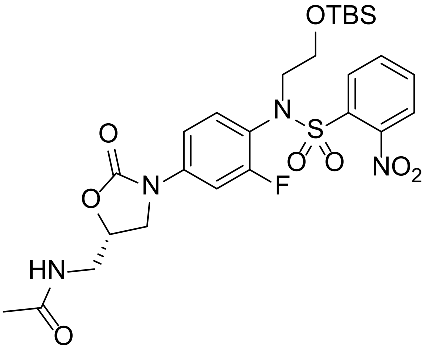


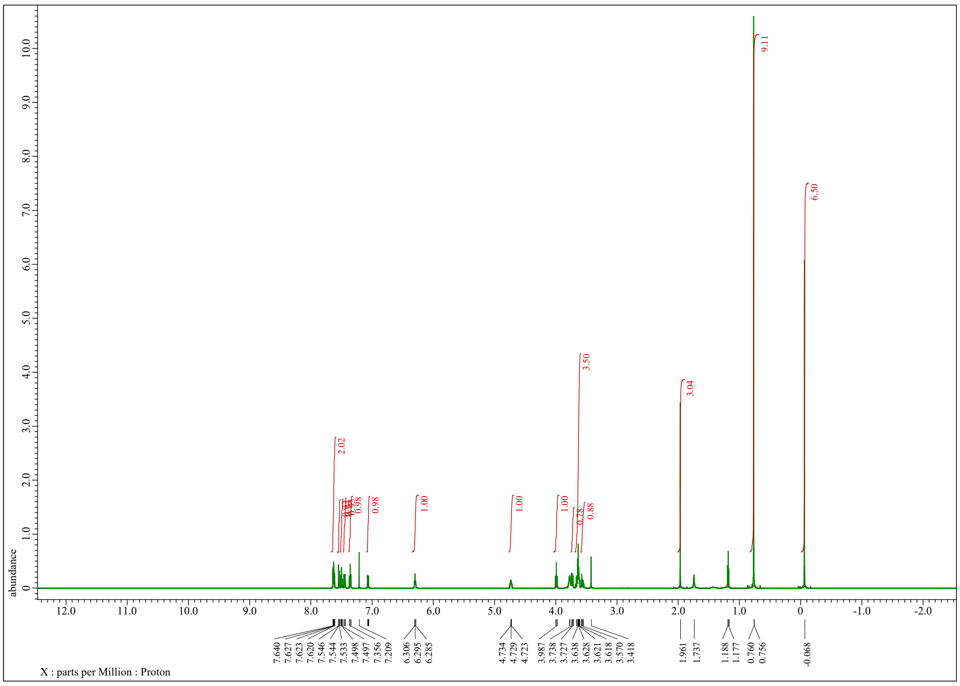


Figure S19. $^{1}$H-NMR spectrum of compound **15**

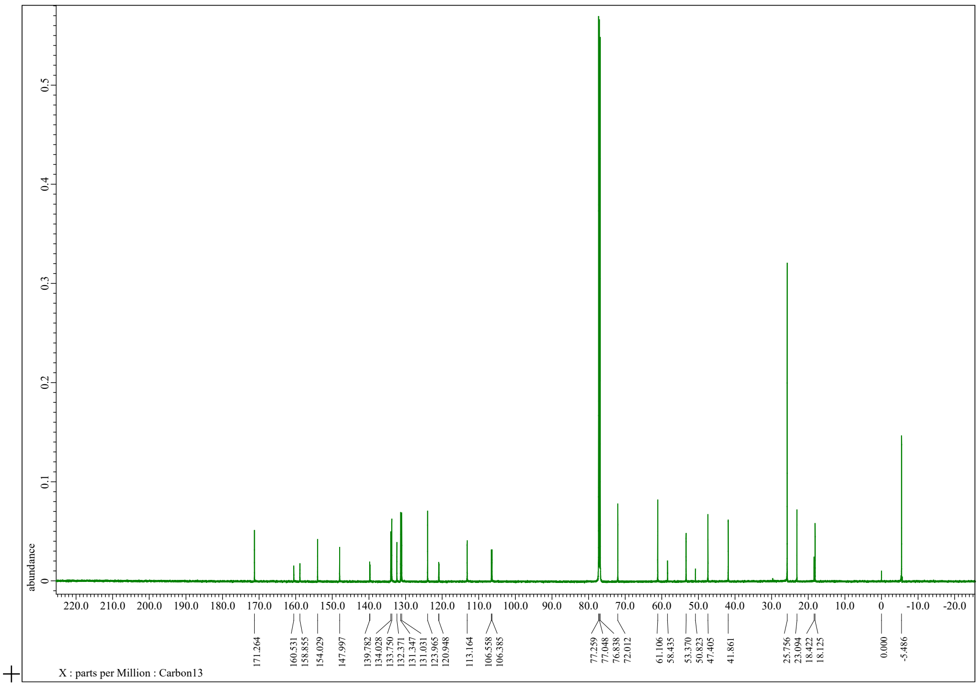


Figure S20. $^{13}$C-NMR spectrum of compound **15**

(*S*)-*N*-((3-(3-Fluoro-4-((*N*-(2-hydroxyethyl)-2-nitrophenyl)sulfonamido)phenyl)-2-oxooxazolidin-5-yl)methyl)acetamide (**16**)

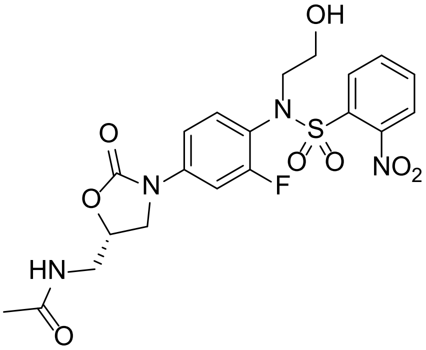


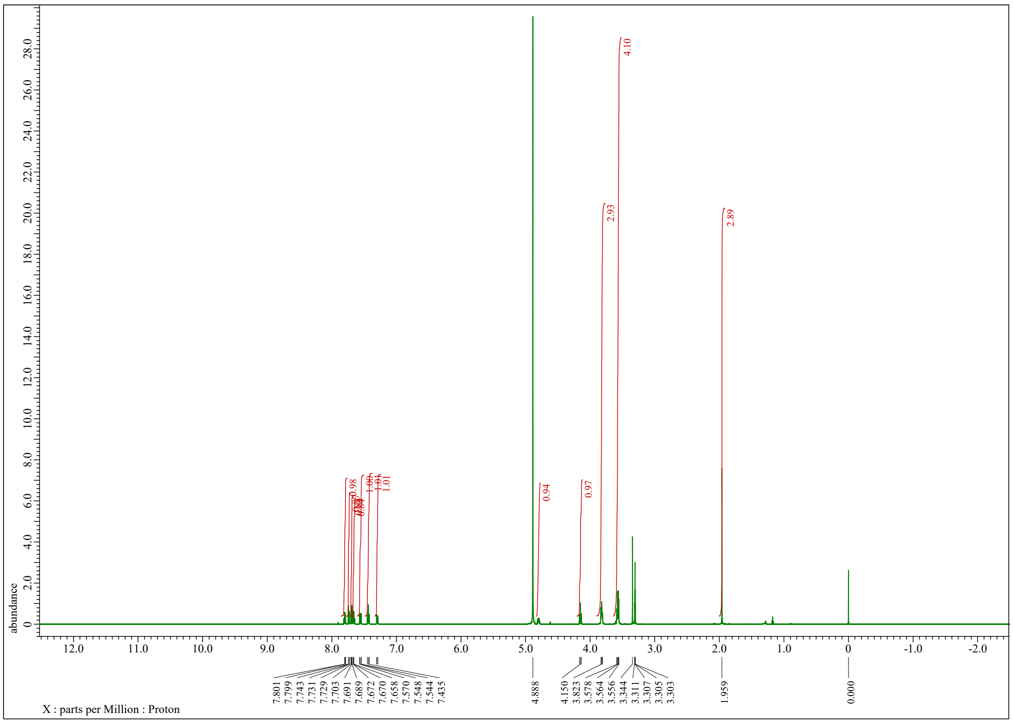


Figure S21. $^{1}$H-NMR spectrum of compound **16**

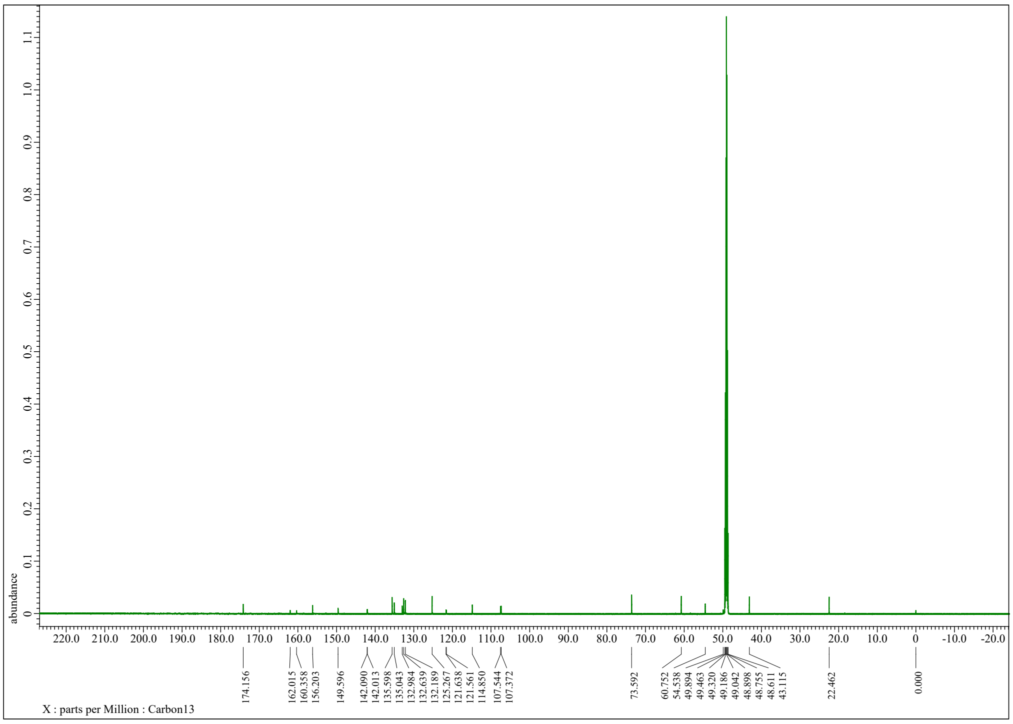


Figure S22. $^{13}$C-NMR spectrum of compound **16**

*tert*-Butyl (*S*)-2-(2-((*N*-(4-(5-(acetamidomethyl)-2-oxooxazolidin-3-yl)-2-fluorophenyl)-2-nitrophenyl)sulfonamido)ethoxy)acetate (**17**)

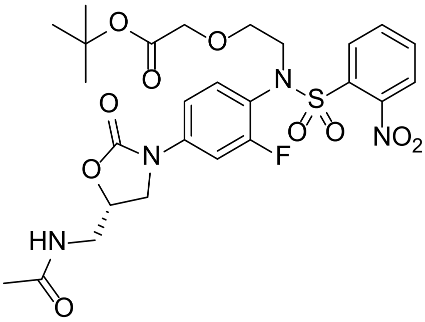


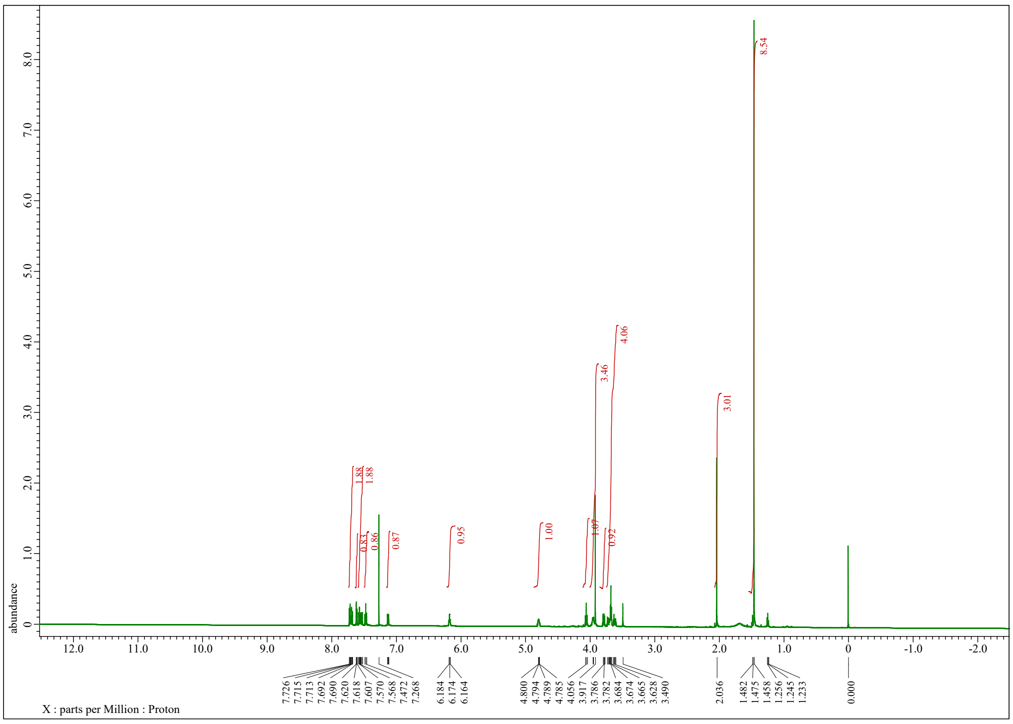


Figure S23. $^{1}$H-NMR spectrum of compound **17**

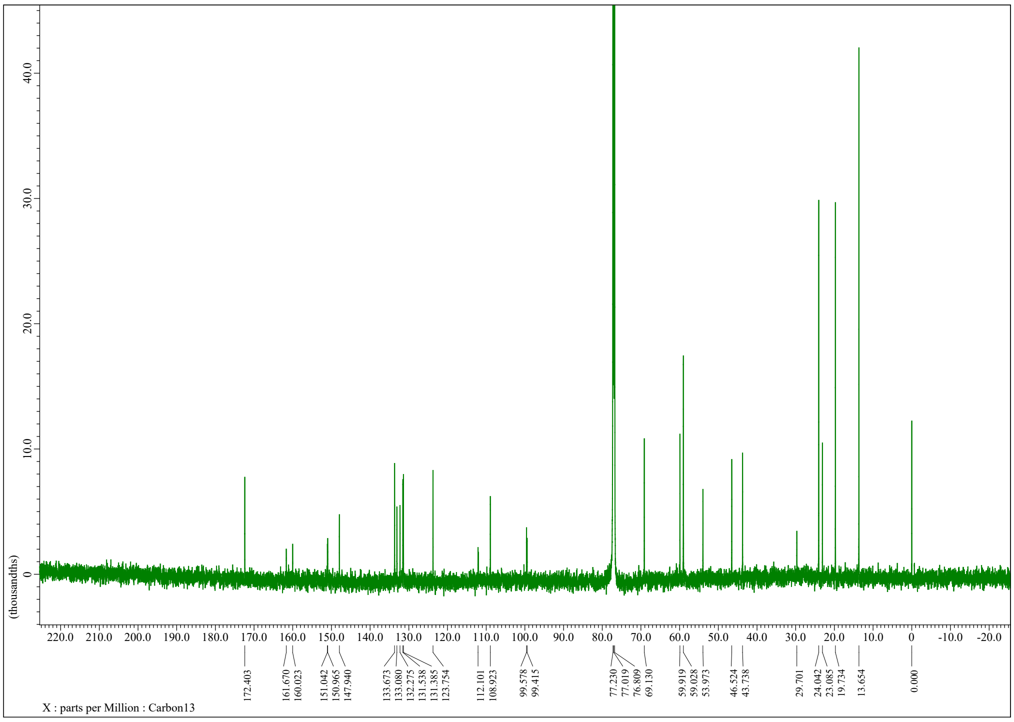


Figure S24. $^{13}$C-NMR spectrum of compound **17**

(*S*)-2-(2-((4-(5-(Acetamidomethyl)-2-oxooxazolidin-3-yl)-2-fluorophenyl)amino)ethoxy)acetic acid (**1**)

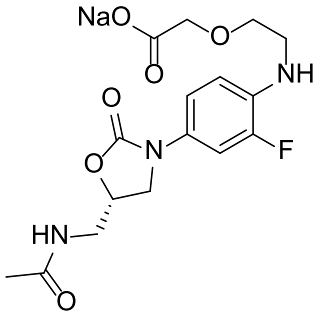


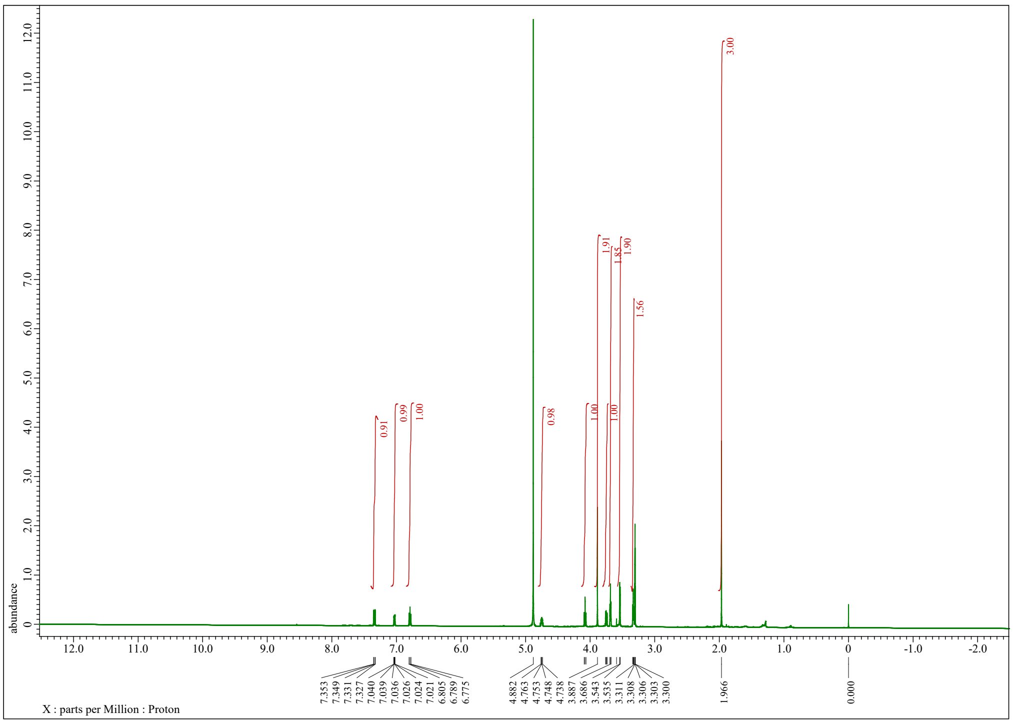

Figure S25. $^{1}$H-NMR spectrum of compound **1**

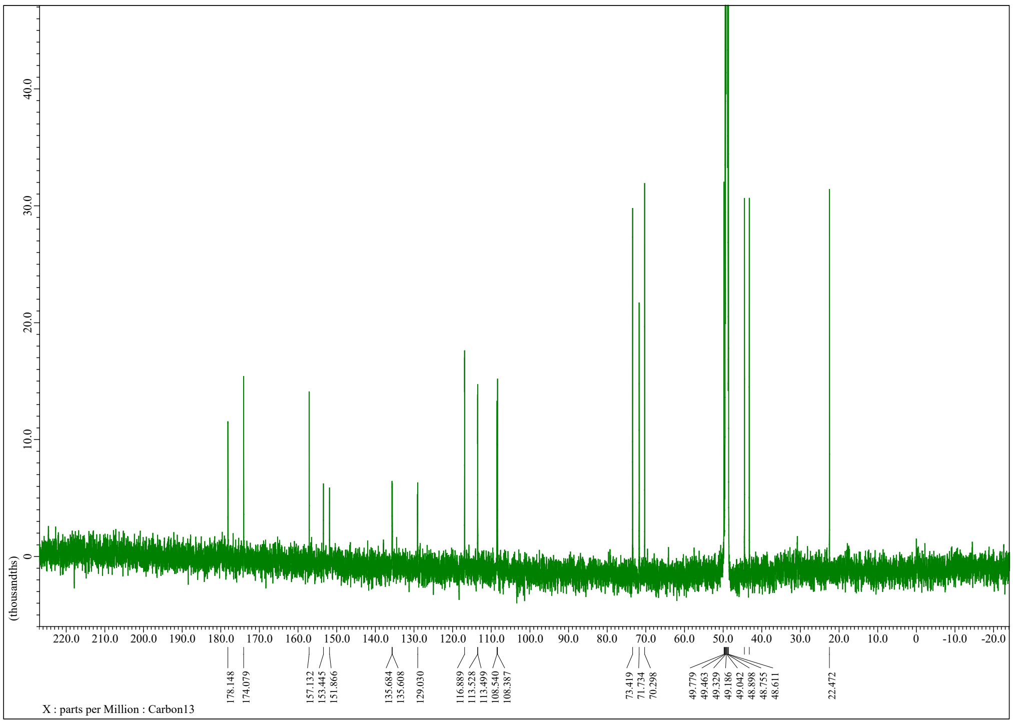

Figure S26. $^{13}$C-NMR spectrum of compound **1**

(*S*)-*N*-((3-(4-((*N*-(2-(Benzyloxy)ethyl)-2-nitrophenyl)sulfonamido)-3-fluorophenyl)-2-oxooxazolidin-5-yl)methyl)acetamide (**18**)

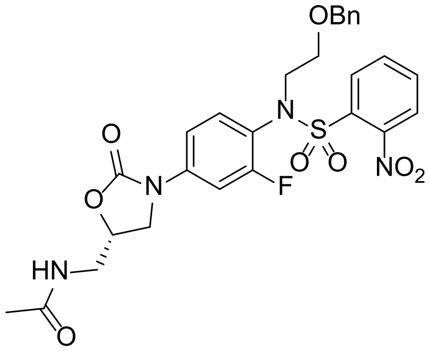


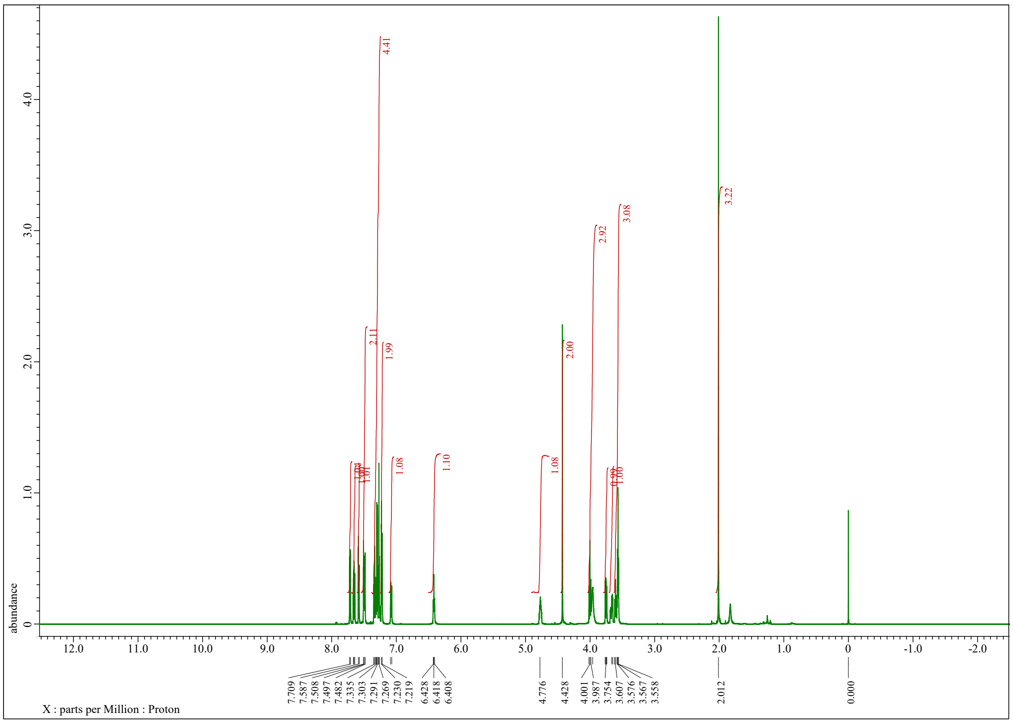


Figure S27. $^{1}$H-NMR spectrum of compound **18**

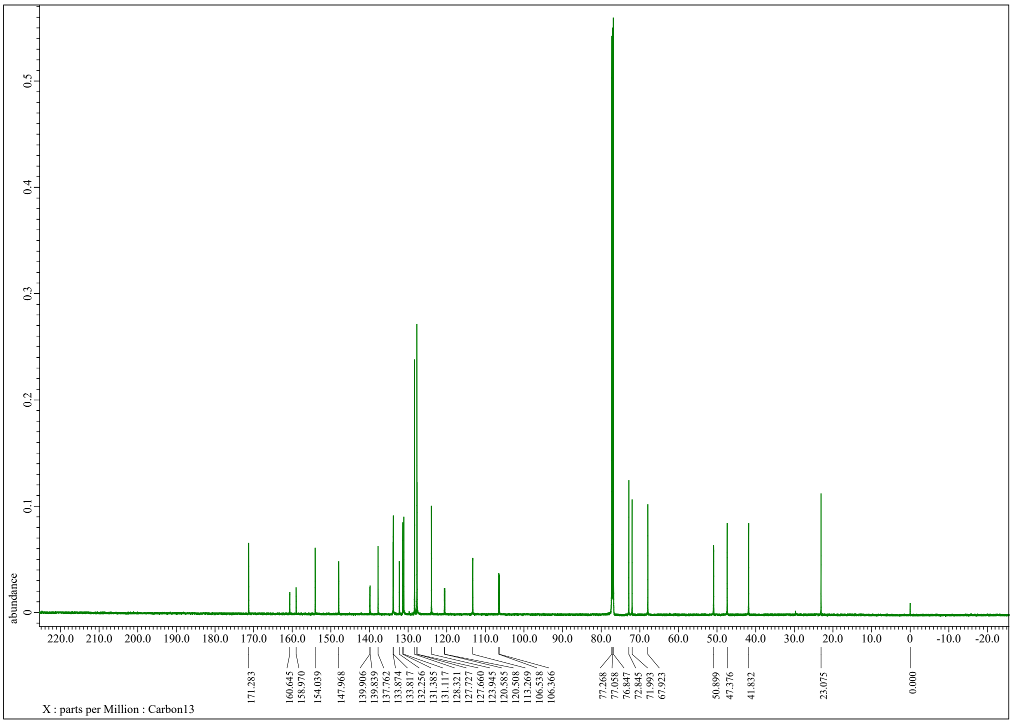


Figure S28. $^{13}$C-NMR spectrum of compound **18**

(*S*)-*N*-((3-(4-((2-(Benzyloxy)ethyl)amino)-3-fluorophenyl)-2-oxooxazolidin-5-yl)methyl)acetamide (**19**)

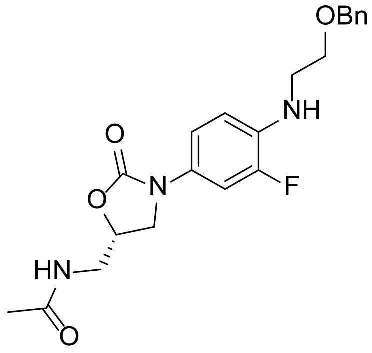


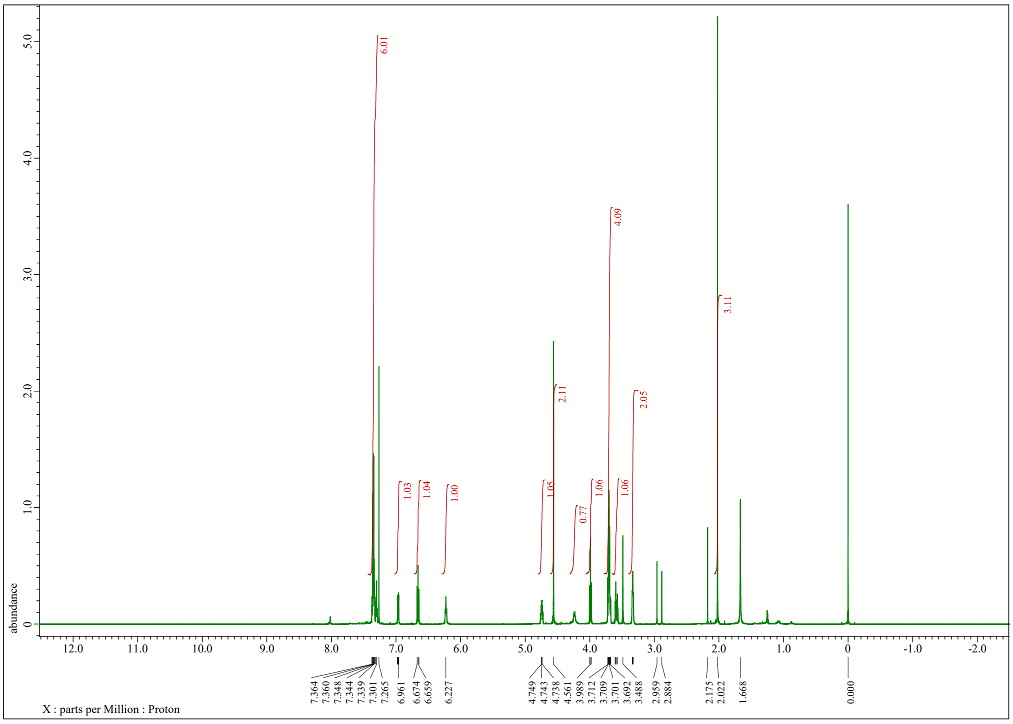

Figure S29. $^{1}$H-NMR spectrum of compound **19**.

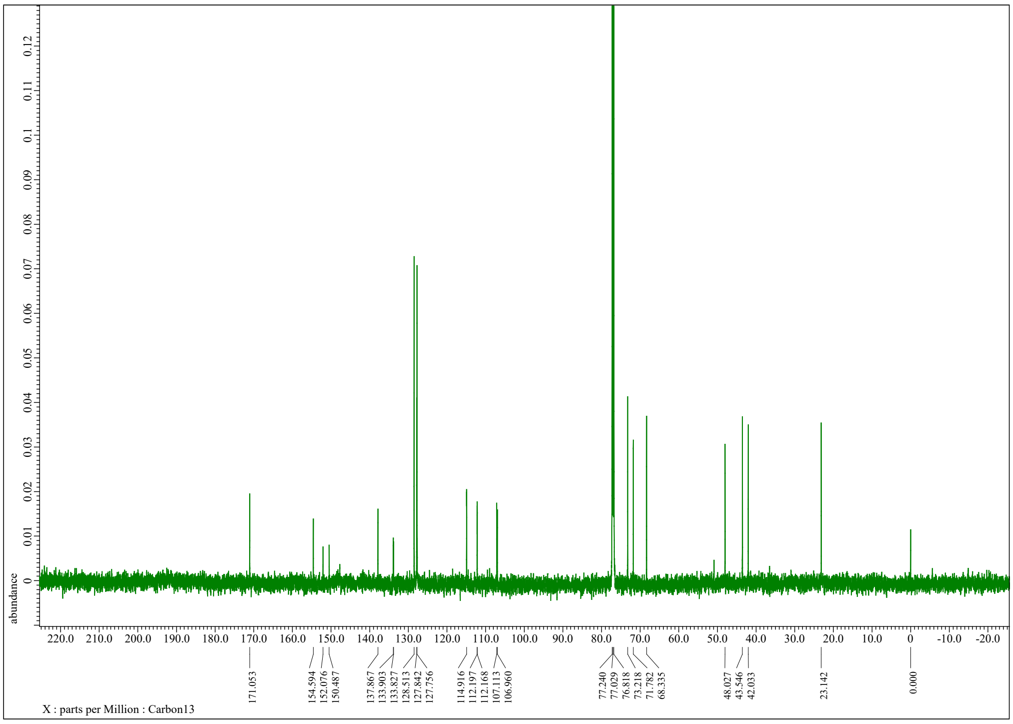

Figure S30. $^{13}$C-NMR spectrum of compound **19**.

Ethyl (*S*)-*N*-(4-(5-(acetamidomethyl)-2-oxooxazolidin-3-yl)-2-fluorophenyl)-*N*-(2-(benzyloxy)ethyl)glycinate (**20**)

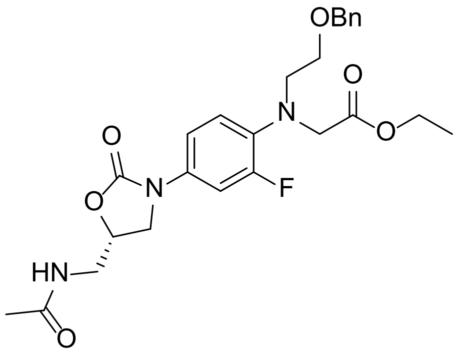

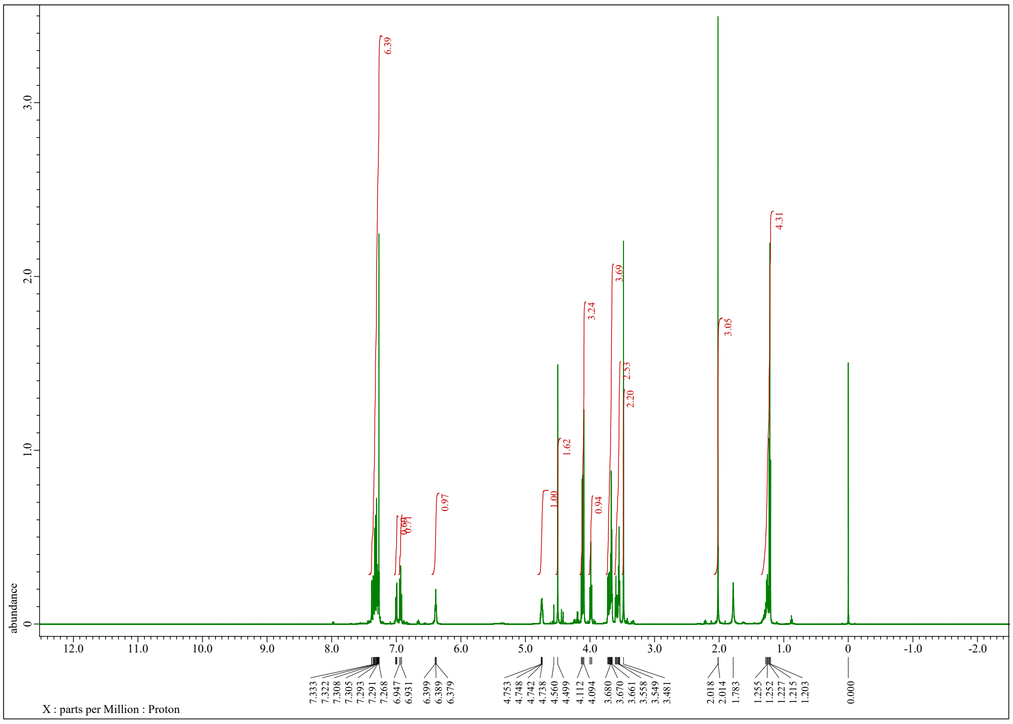


Figure S31. $^{1}$H-NMR spectrum of compound **20**.

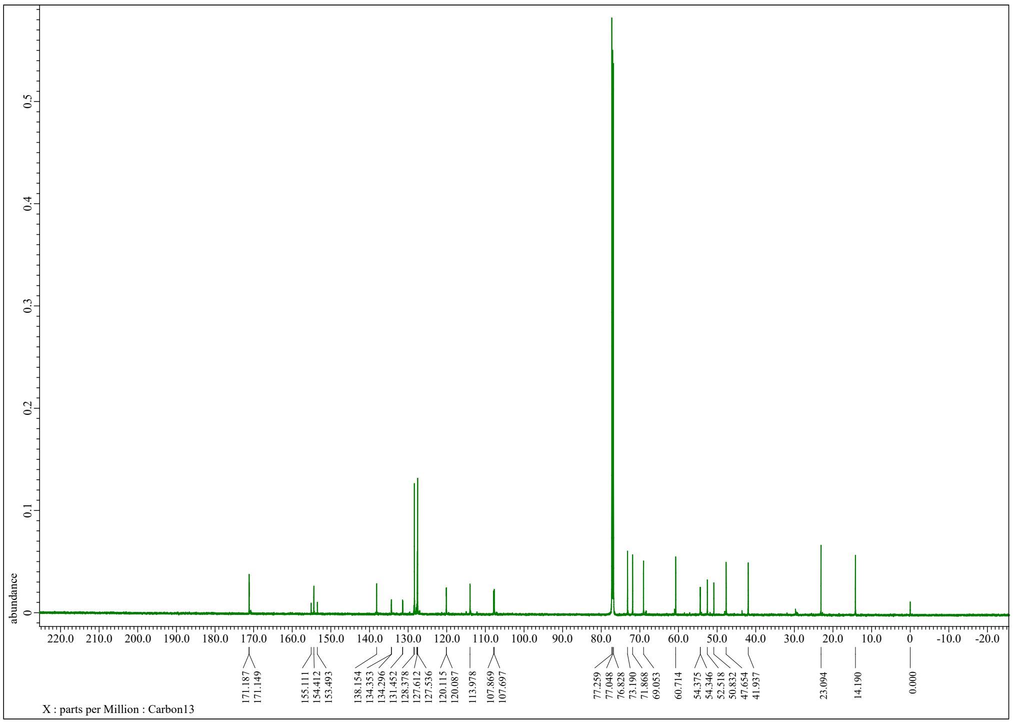


Figure S32. $^{13}$C-NMR spectrum of compound **20**.

Ethyl (*S*)-*N*-(4-(5-(acetamidomethyl)-2-oxooxazolidin-3-yl)-2-fluorophenyl)-*N*-(2-hydroxyethyl)glycinate (**21**)

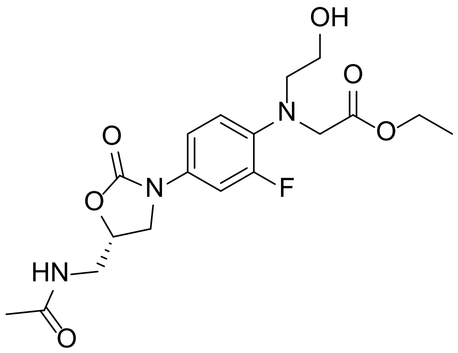


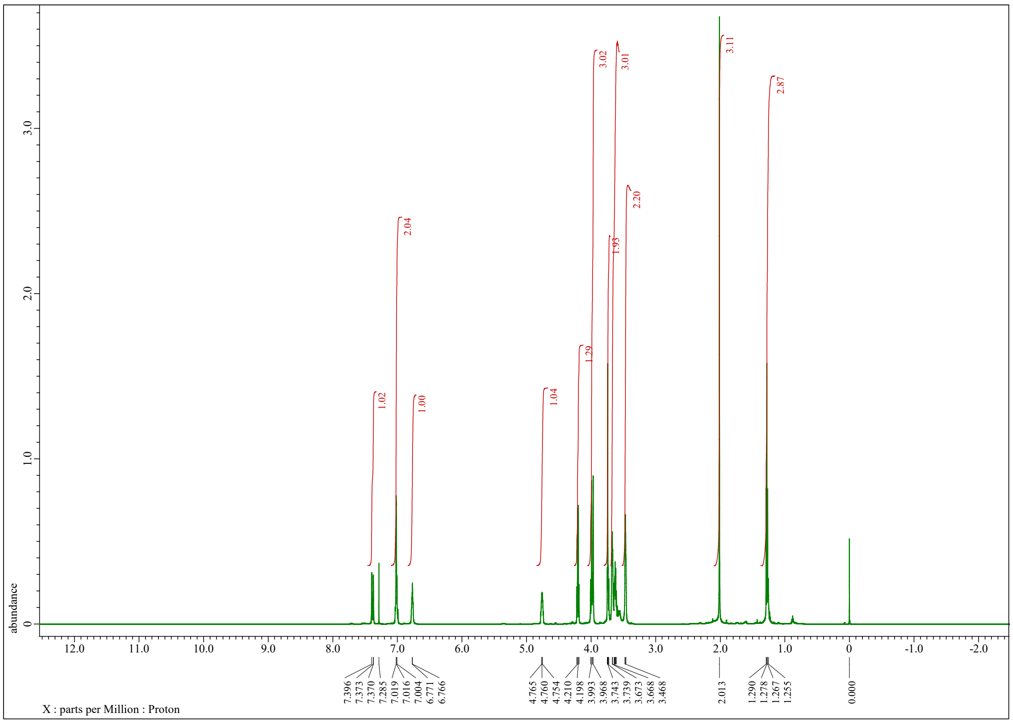


Figure S33. $^{1}$H-NMR spectrum of compound **21**.

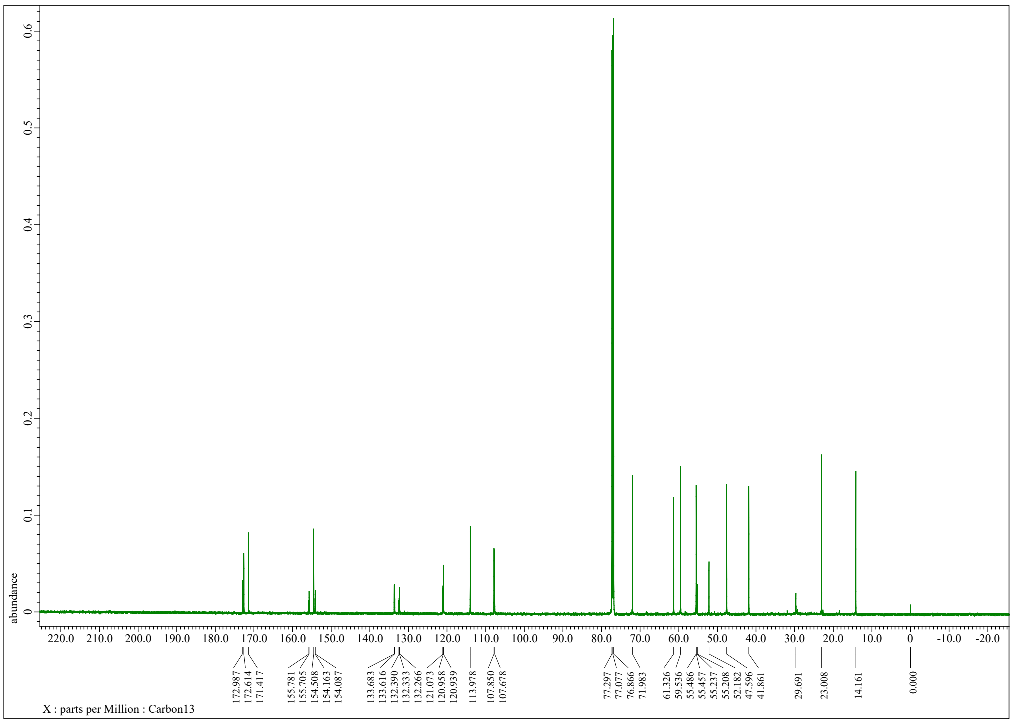


Figure S34. $^{13}$C-NMR spectrum of compound **21**.

(*S*)-*N*-(4-(5-(Acetamidomethyl)-2-oxooxazolidin-3-yl)-2-fluorophenyl)-*N*-(2-hydroxyethyl)glycinate (**2**)

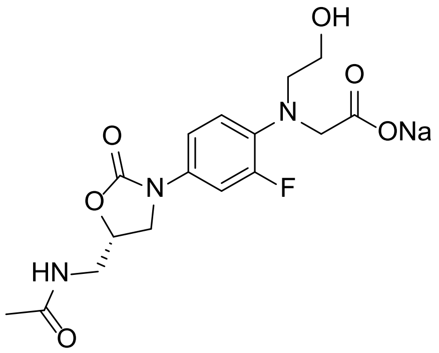


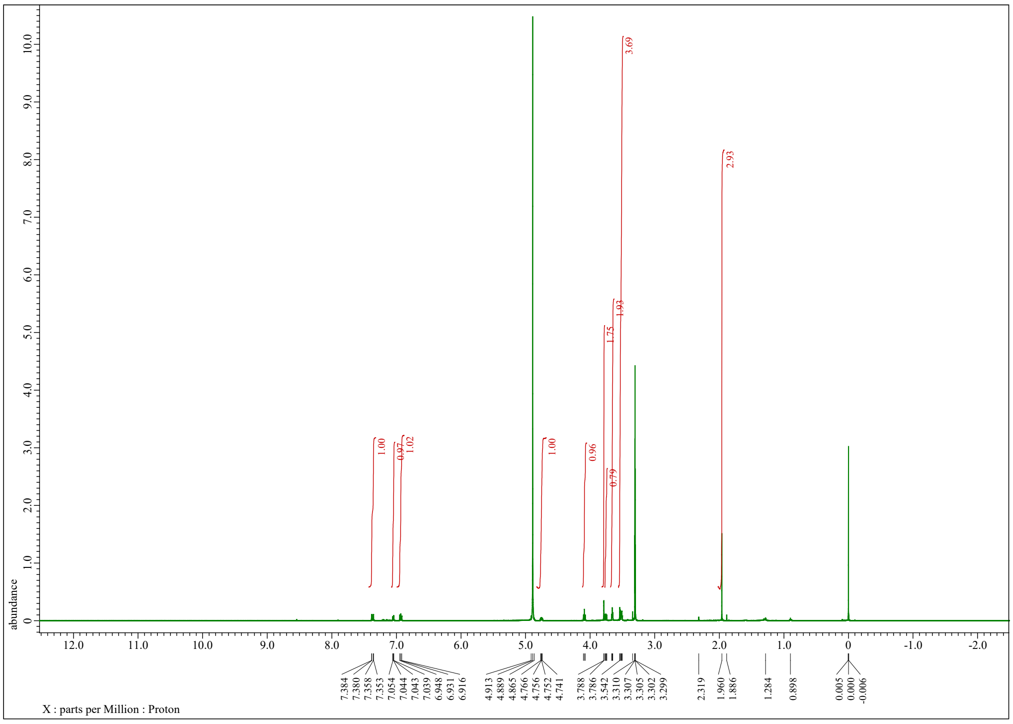


Figure S35. $^{1}$H-NMR spectrum of compound **2**.

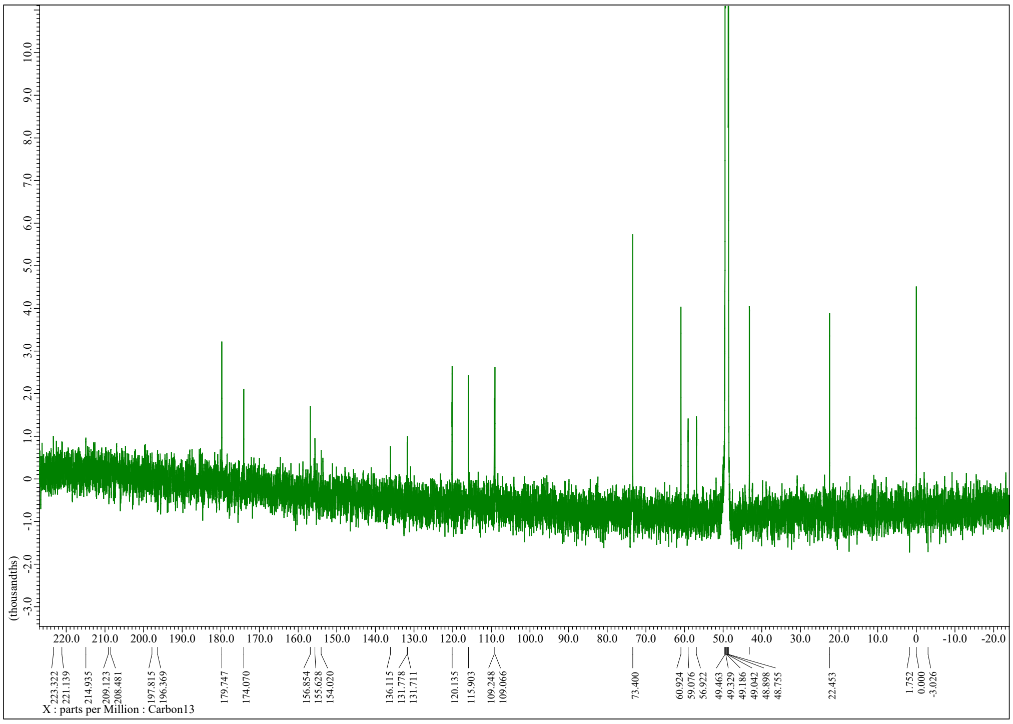


Figure S36. $^{13}$C-NMR spectrum of compound **2**.

(*S*)-*N*-((3-(3-Fluoro-4-((2-hydroxyethyl)amino)phenyl)-2-oxooxazolidin-5-yl)methyl)acetamide (**3**)

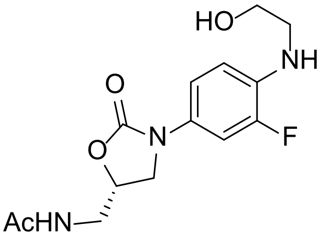


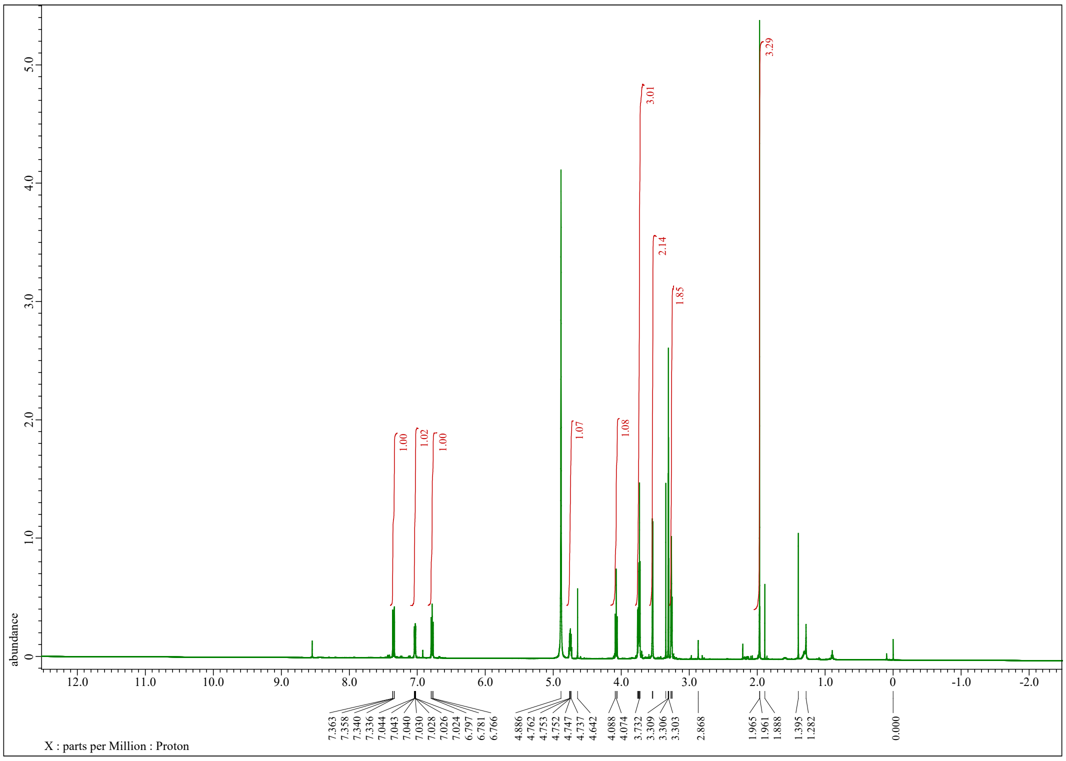


Figure S37. $^{1}$H-NMR spectrum of compound **3**.

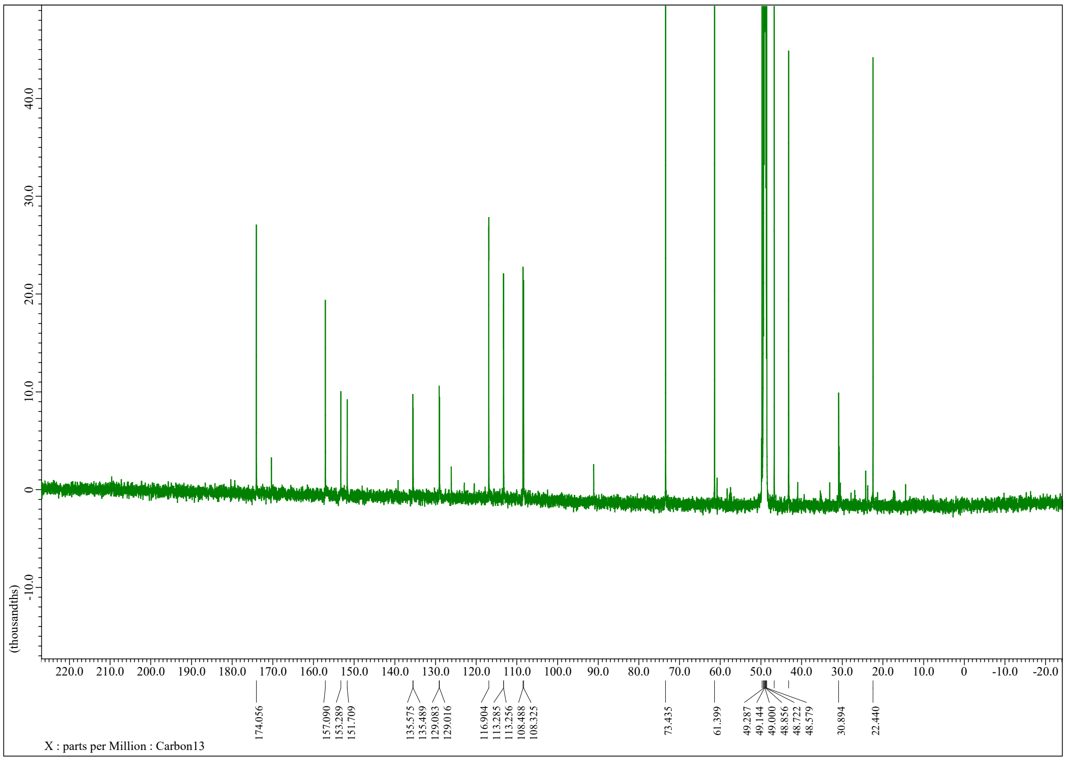


Figure S38. $^{13}$C-NMR spectrum of compound **3**.

Ethyl (*S*)-*N*-(4-(5-(acetamidomethyl)-2-oxooxazolidin-3-yl)-2-fluorophenyl)-*N*-((4-nitrophenyl)sulfonyl)glycinate (**22**)

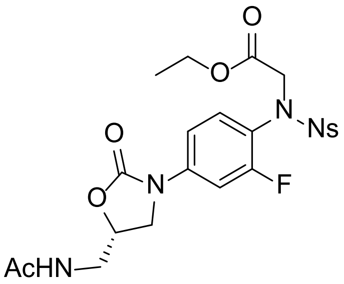


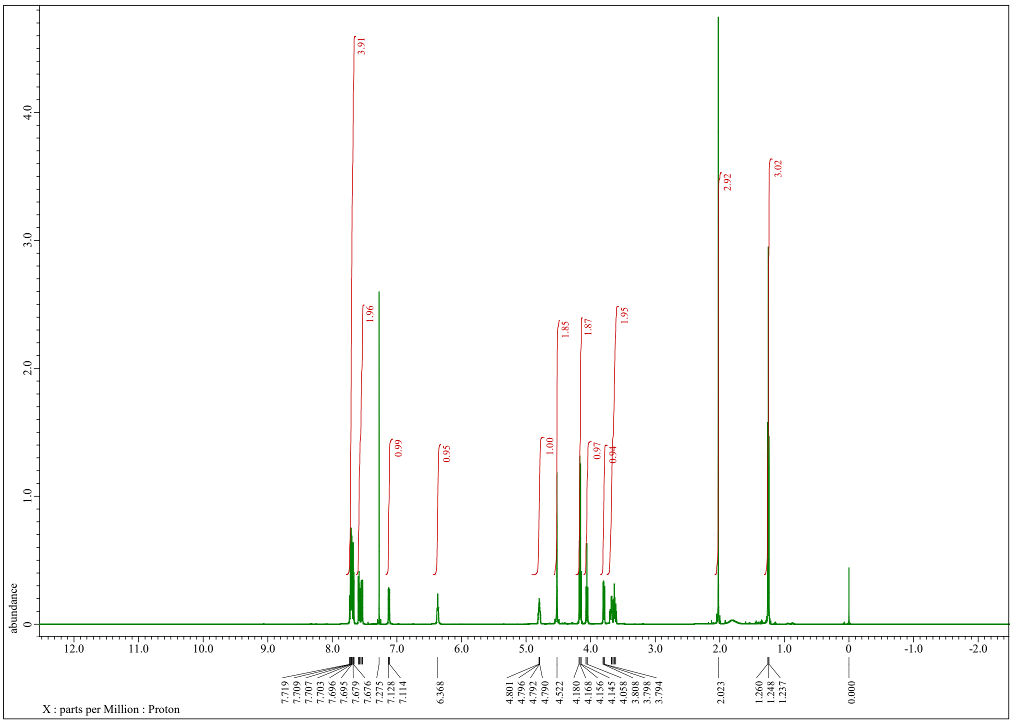


Figure S39. $^{1}$H-NMR spectrum of compound **22**.

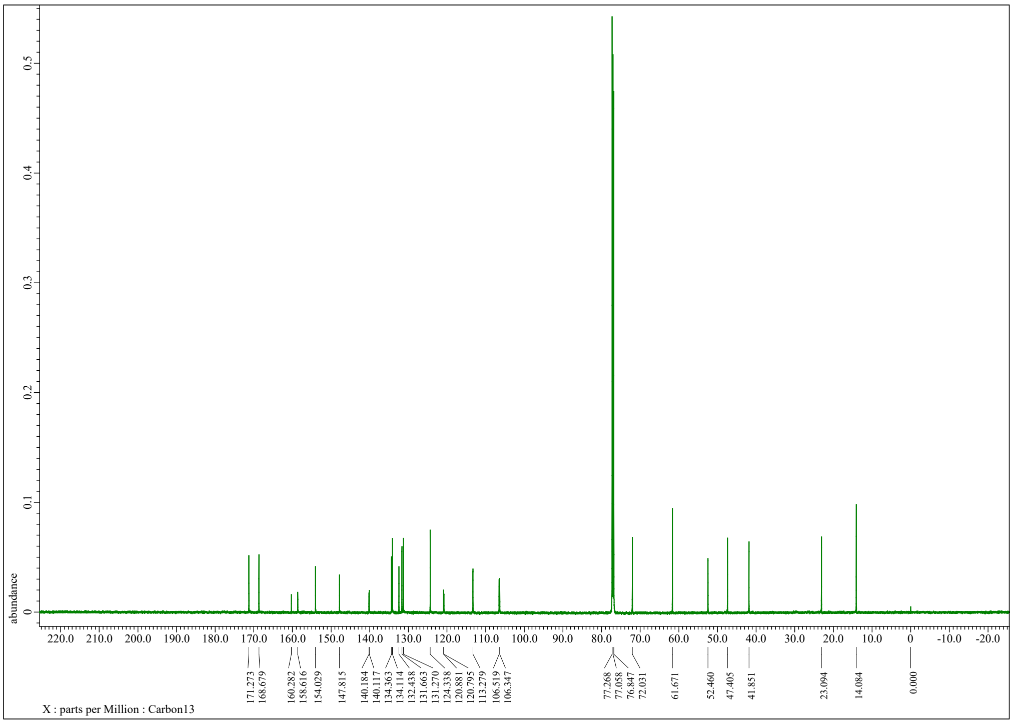


Figure S40. $^{13}$C-NMR spectrum of compound **22**.

Ethyl (*S*)-(4-(5-(acetamidomethyl)-2-oxooxazolidin-3-yl)-2-fluorophenyl)glycinate (**23**)

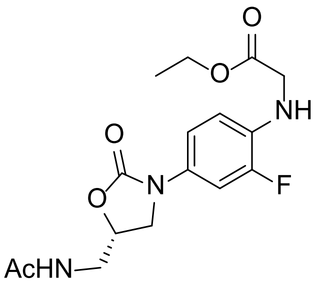


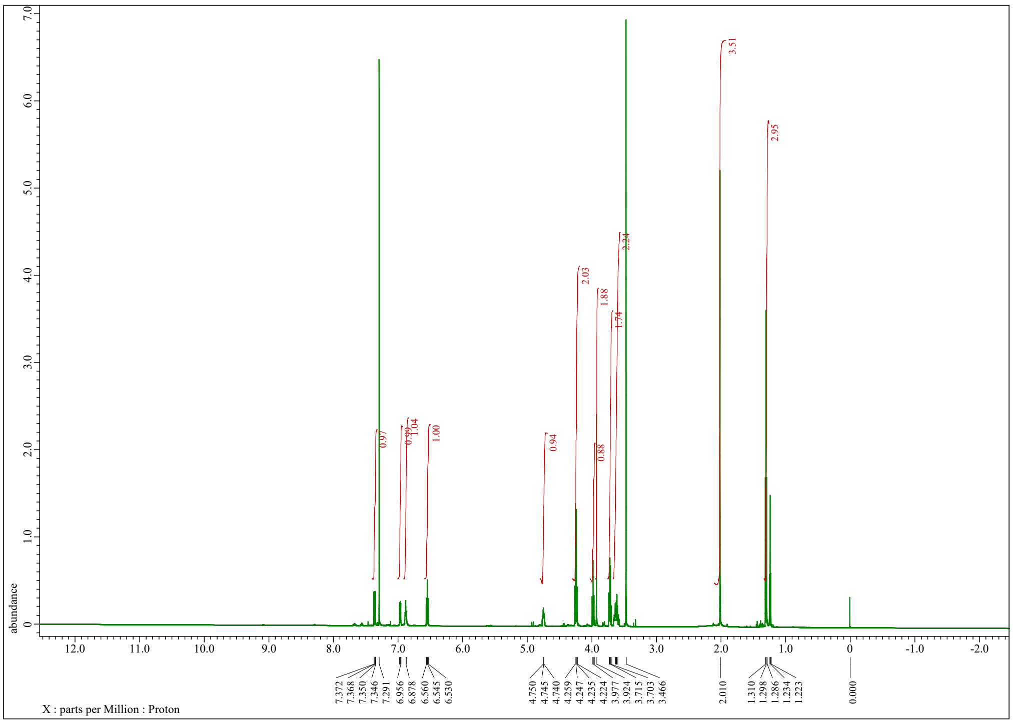

Figure S41. $^1$H-NMR spectrum of compound **23**.

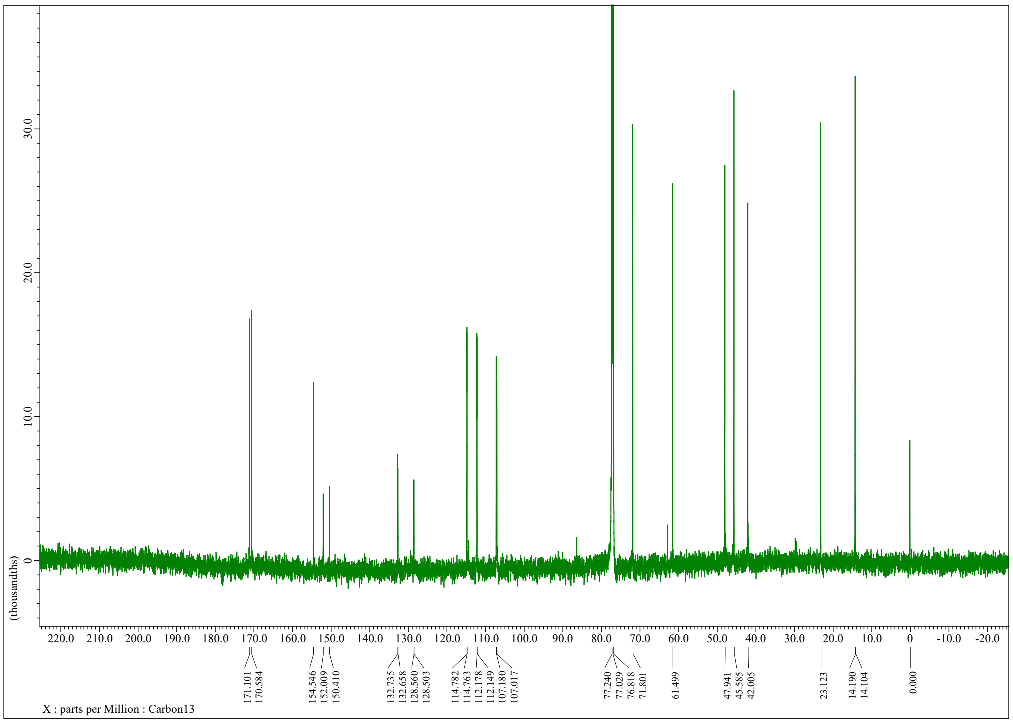

Figure S42. $^{13}$C-NMR spectrum of compound **23**.

(*S*)-(4-(5-(acetamidomethyl)-2-oxooxazolidin-3-yl)-2-fluorophenyl)glycinate (**4**)

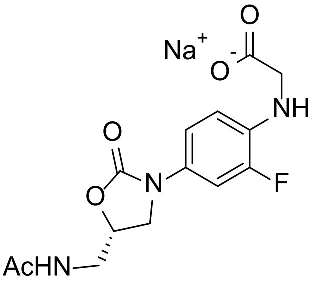


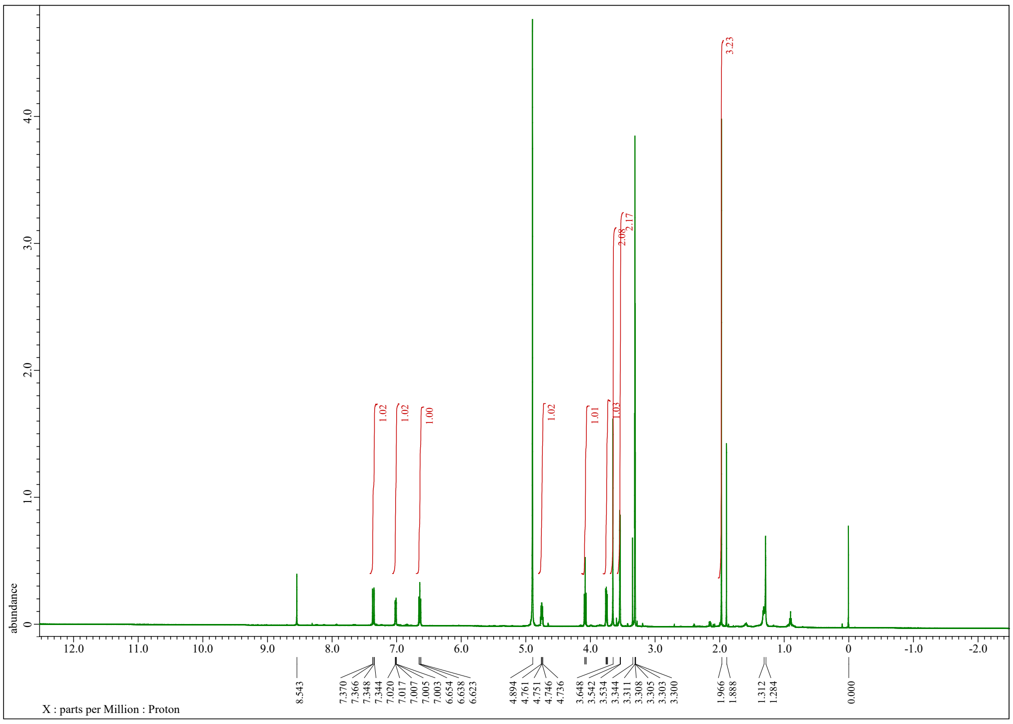


Figure S43. $^{1}$H-NMR spectrum of compound **4**.

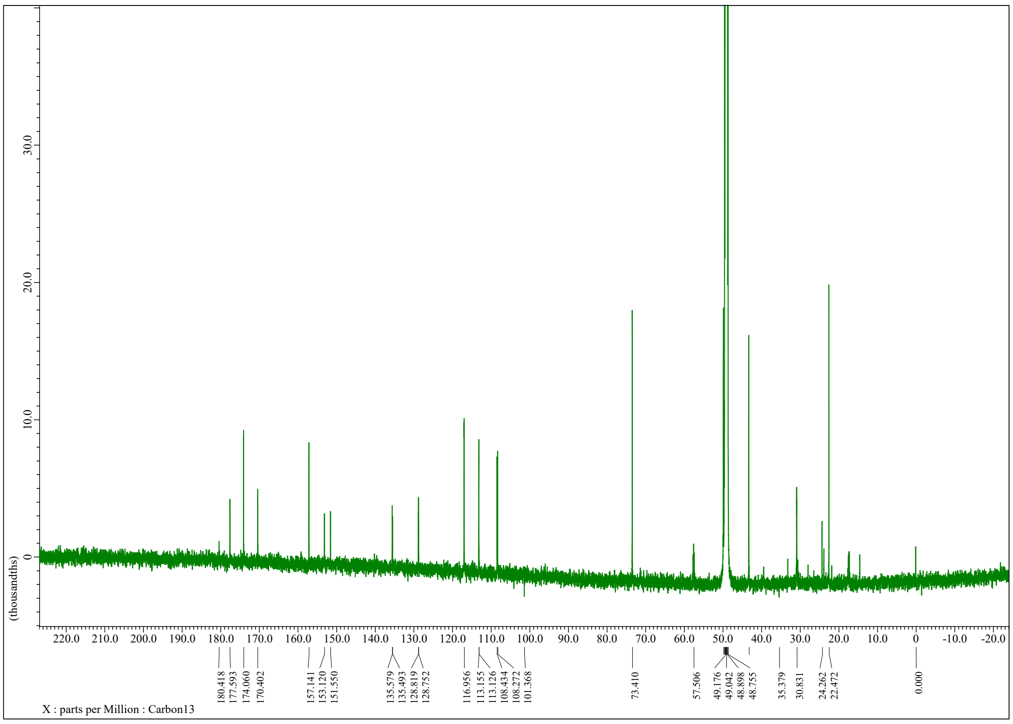


Figure S44. $^{13}$C-NMR spectrum of compound **4**.
